# Supplementary figures and images for: Regulation of lipid metabolism by APOE4 in intrahepatic cholangiocarcinoma via the enhancement of ABCA1 membrane expression
Source: PeerJ. 2024 Jan 22;12:e16740. doi: 10.7717/peerj.16740 (PMC10809977; doi:10.7717/peerj.16740)

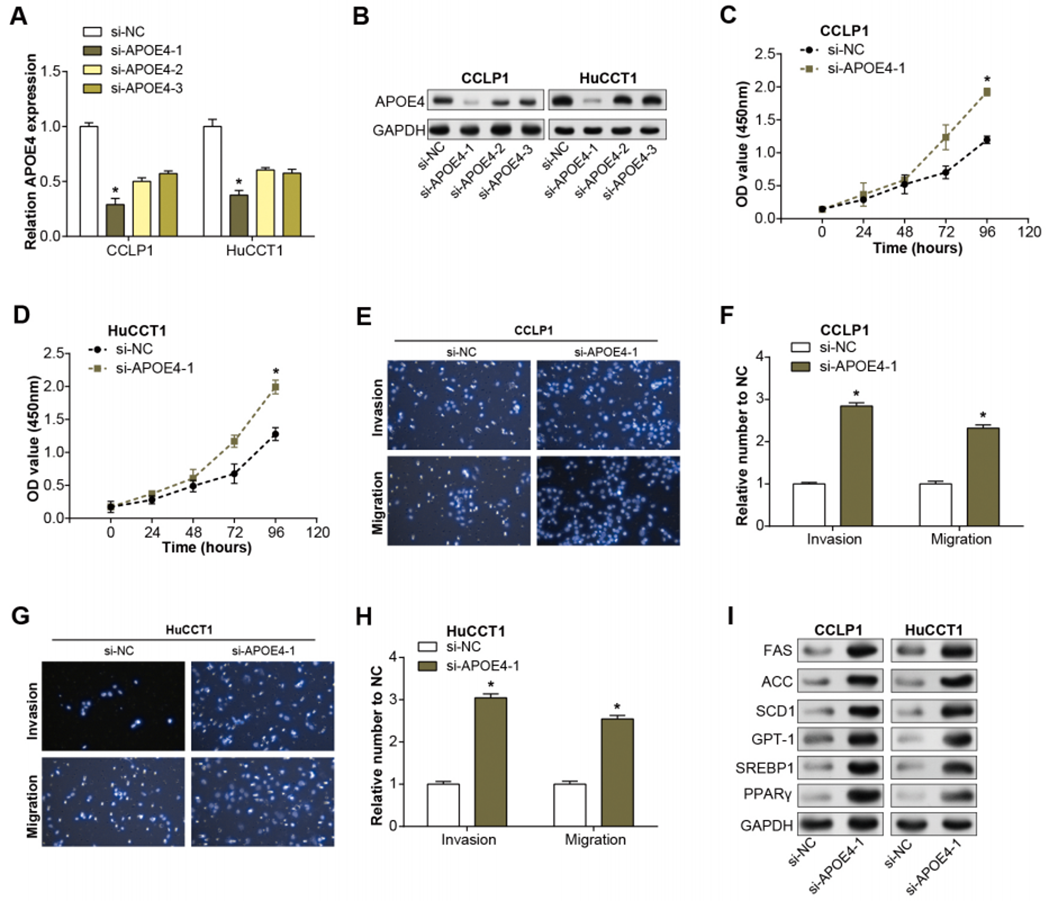

Supplement: Figure S1 [file peerj-12-16740-s001.png]

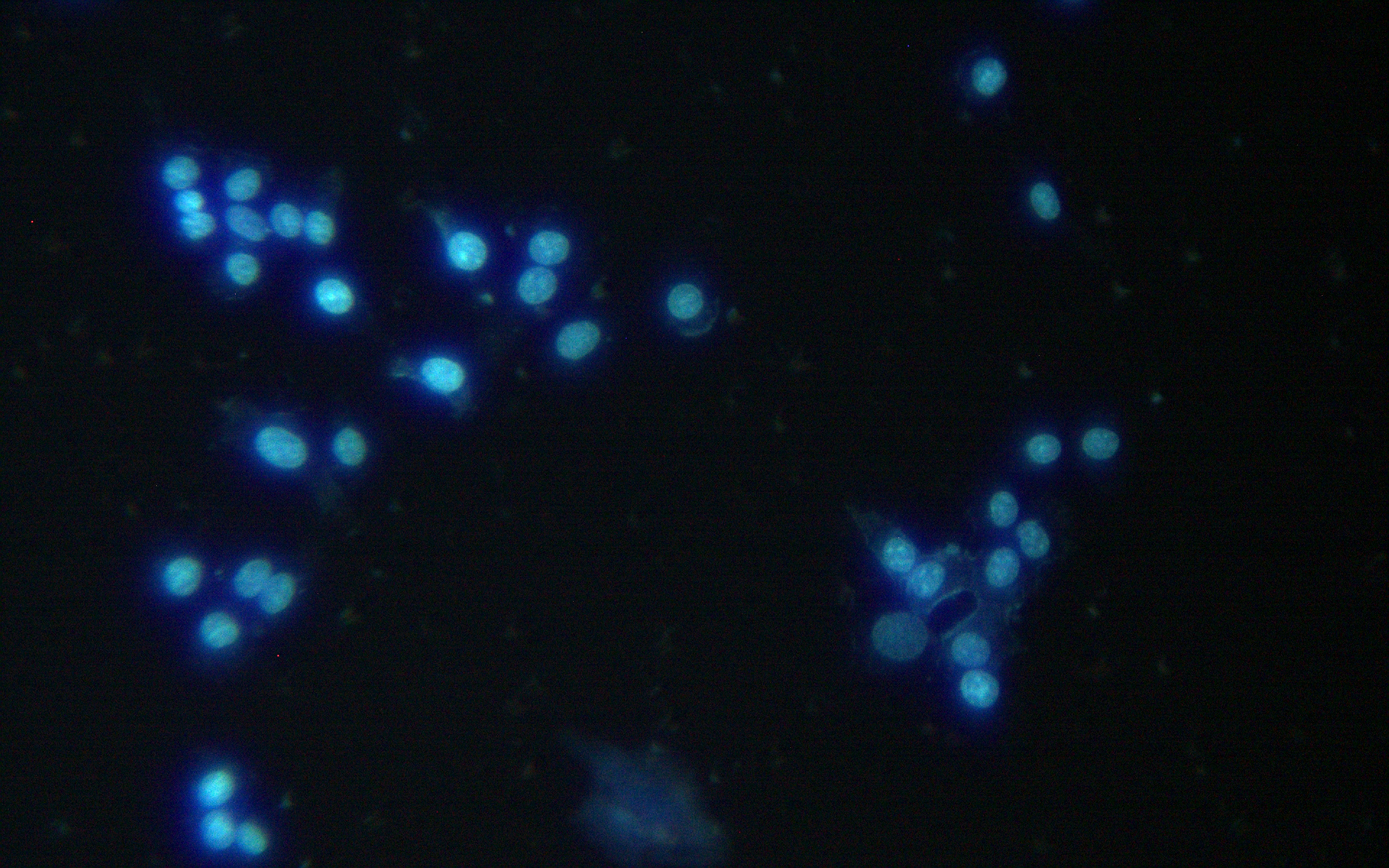

Supplement: Supplemental Information 5 [file peerj-12-16740-s005.zip › transwell-invasion-CCLP1/Figure 4F_over-APOE4_CCLP1_Invasion.tif]

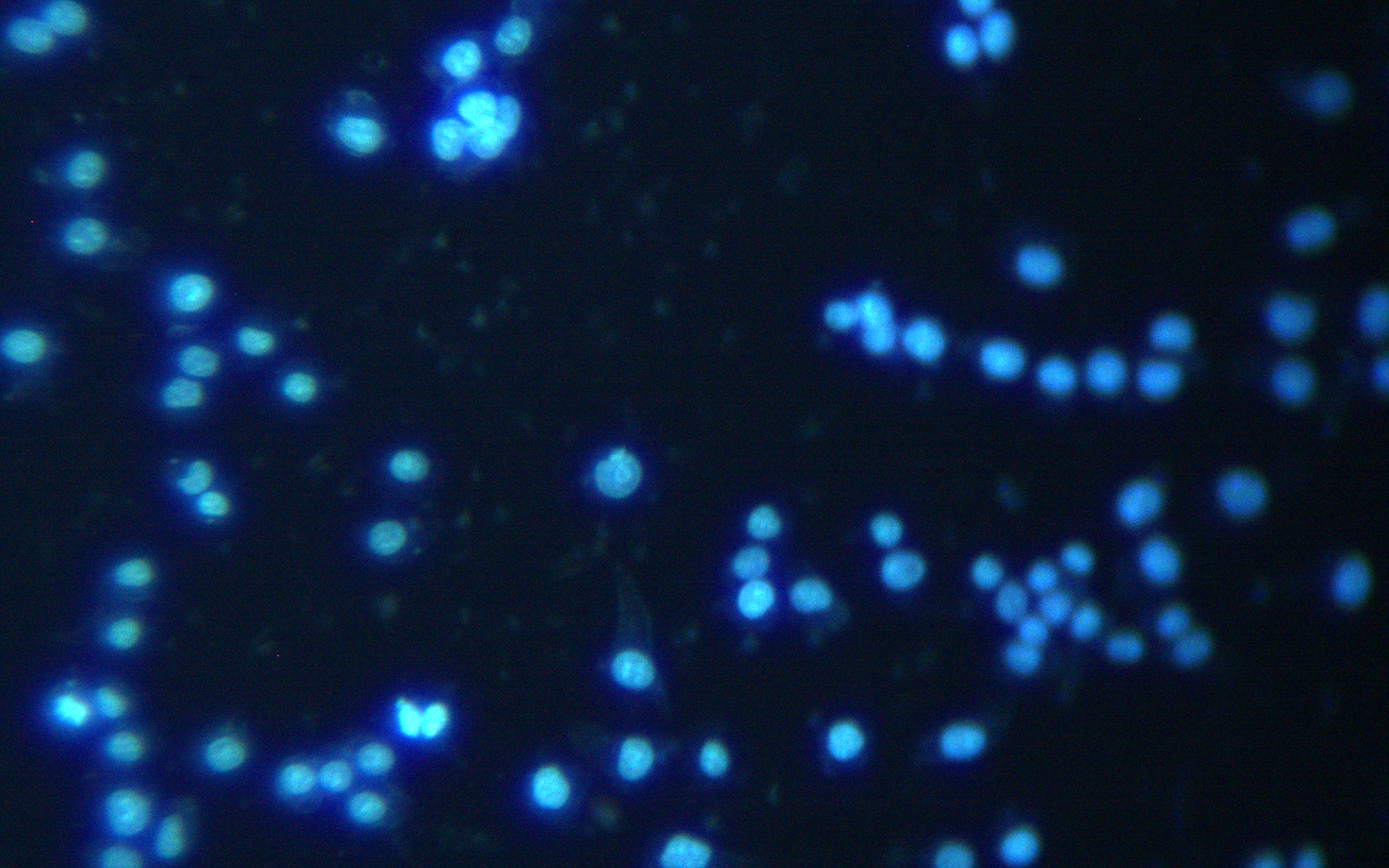

Supplement: Supplemental Information 5 [file peerj-12-16740-s005.zip › transwell-invasion-CCLP1/Figure 4F_over-NC_CCLP1_Invasion.tif]

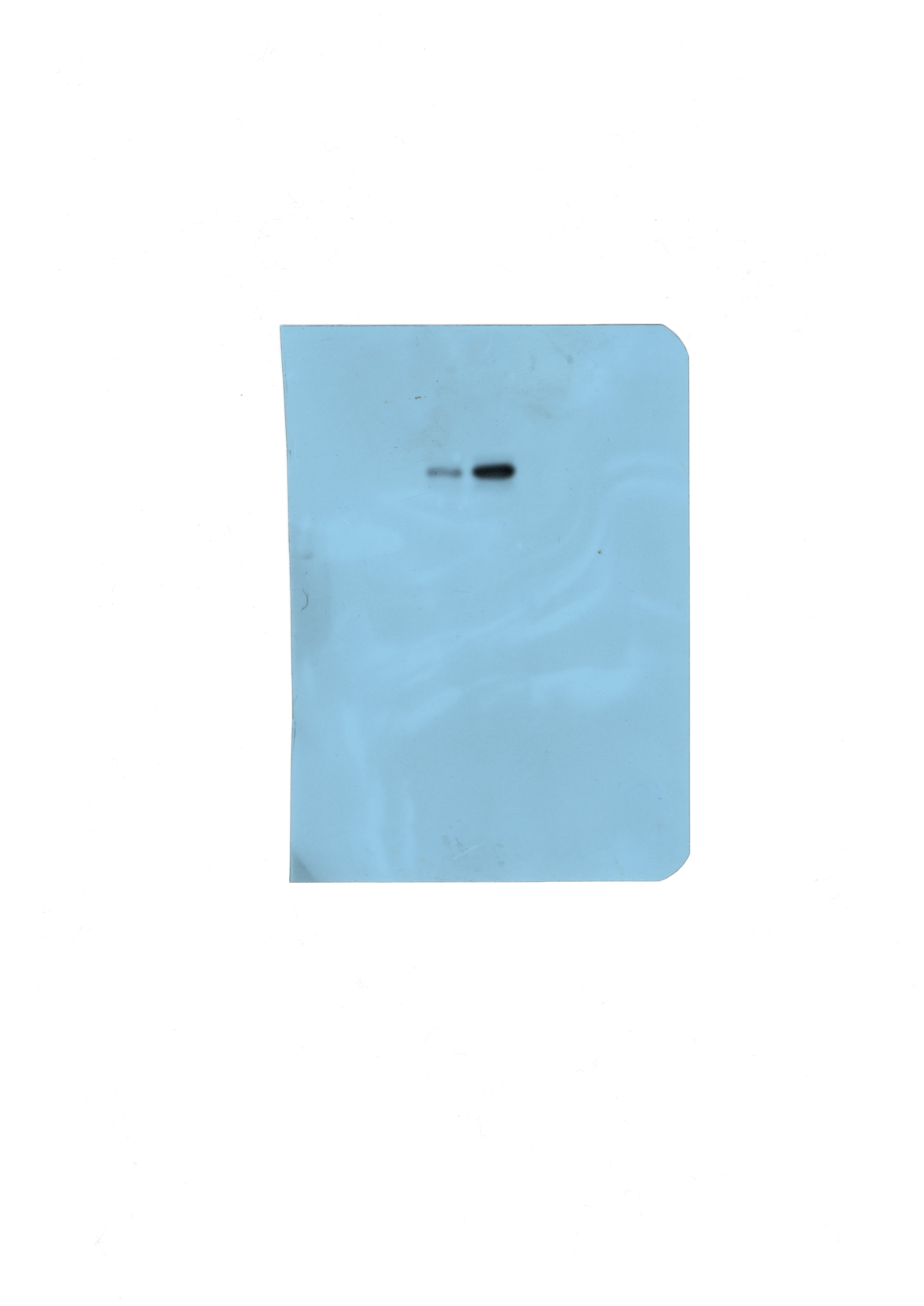

Supplement: Supplemental Information 7 [file peerj-12-16740-s007.zip › Figure 4C-CCLP1/Figure 4C_APOE4_CCLP1.tif]

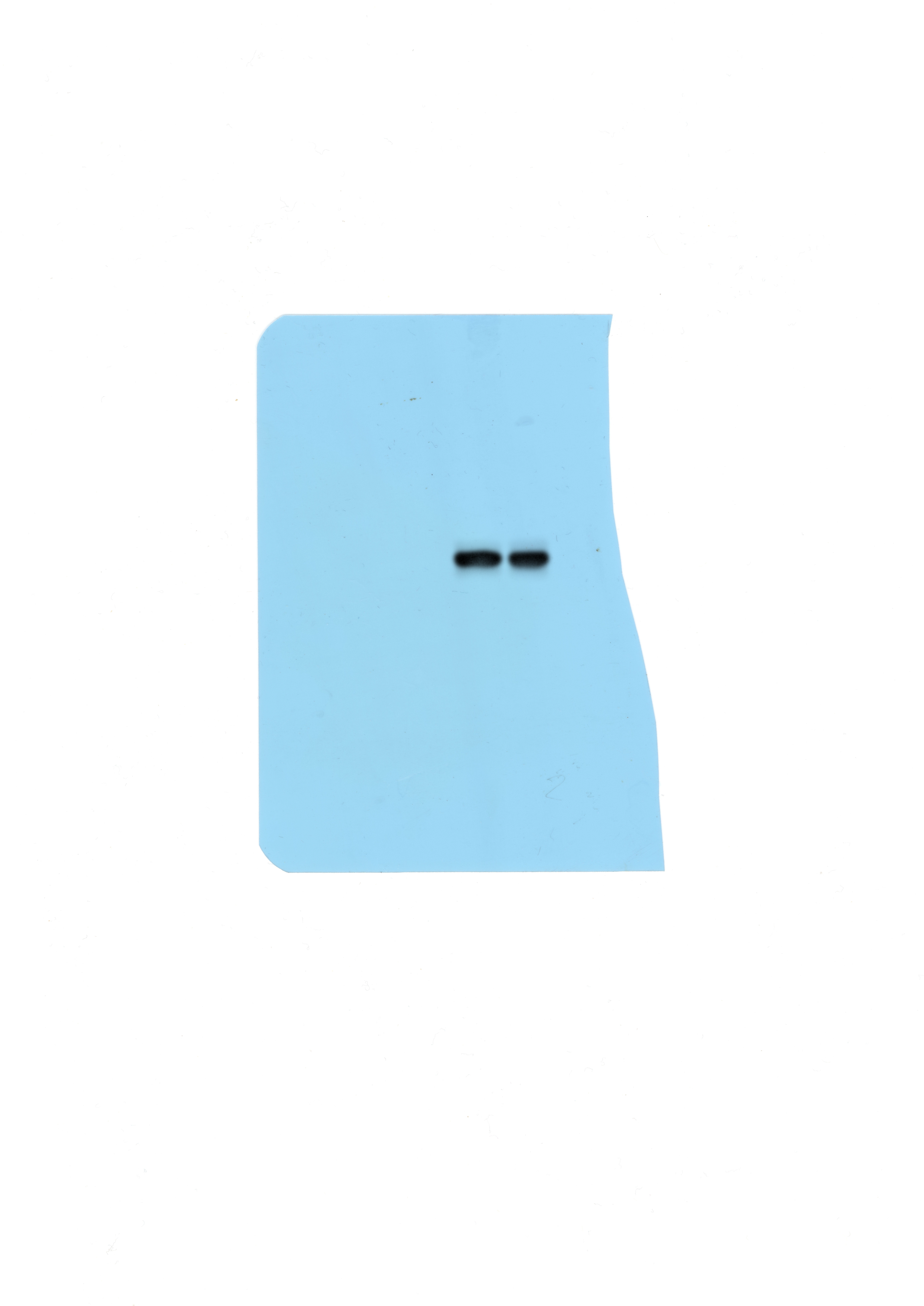

Supplement: Supplemental Information 7 [file peerj-12-16740-s007.zip › Figure 4C-CCLP1/Figure 4C_GAPDH_CCLP1.tif]

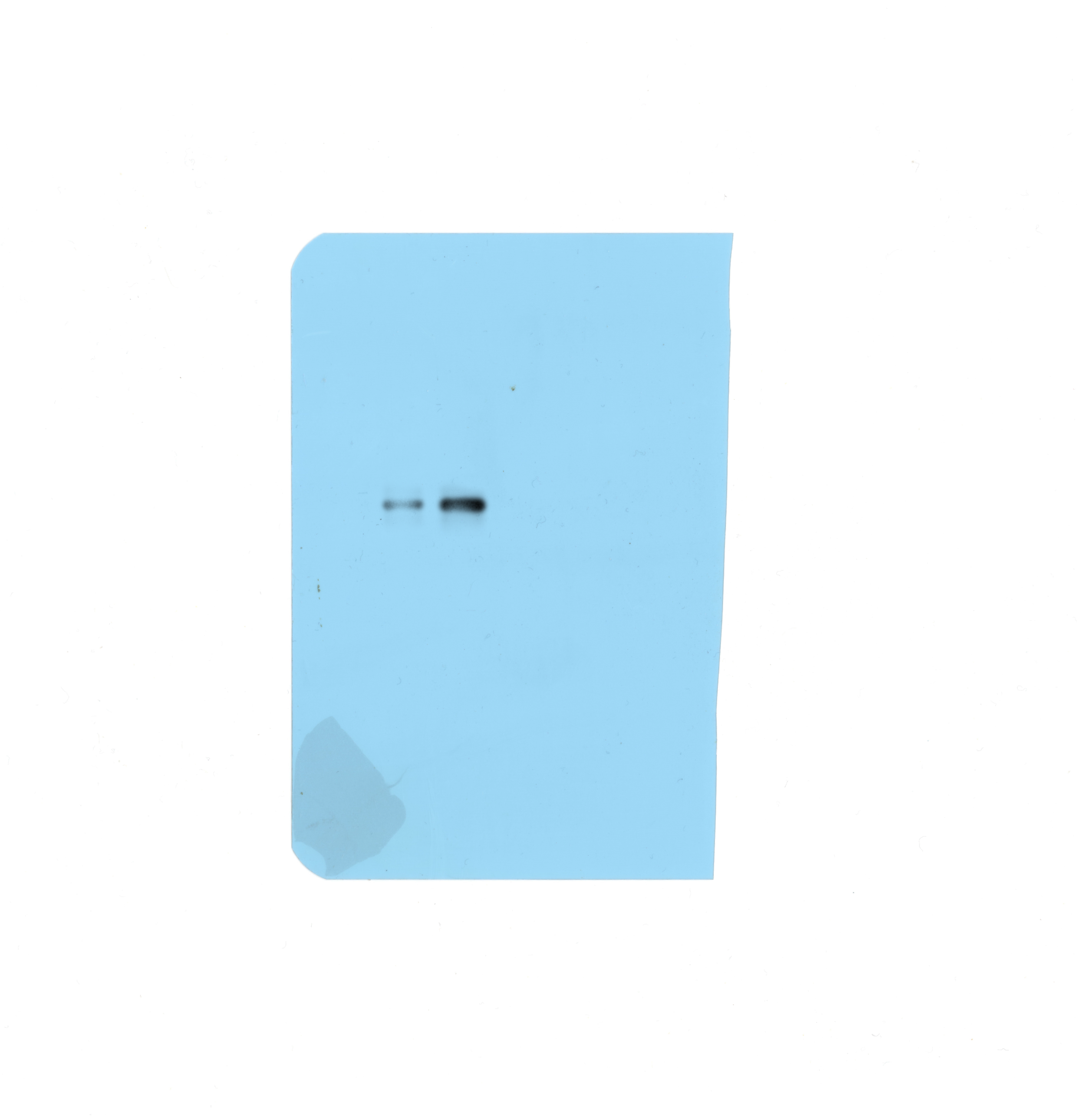

Supplement: Supplemental Information 8 [file peerj-12-16740-s008.zip › WB-Fugure 6/Figure 6B_ABCA1_CCLP1.tif]

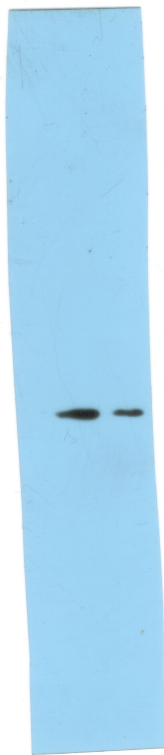

Supplement: Supplemental Information 8 [file peerj-12-16740-s008.zip › WB-Fugure 6/Figure 6B_ABCA1_HuCCT1.pdf]

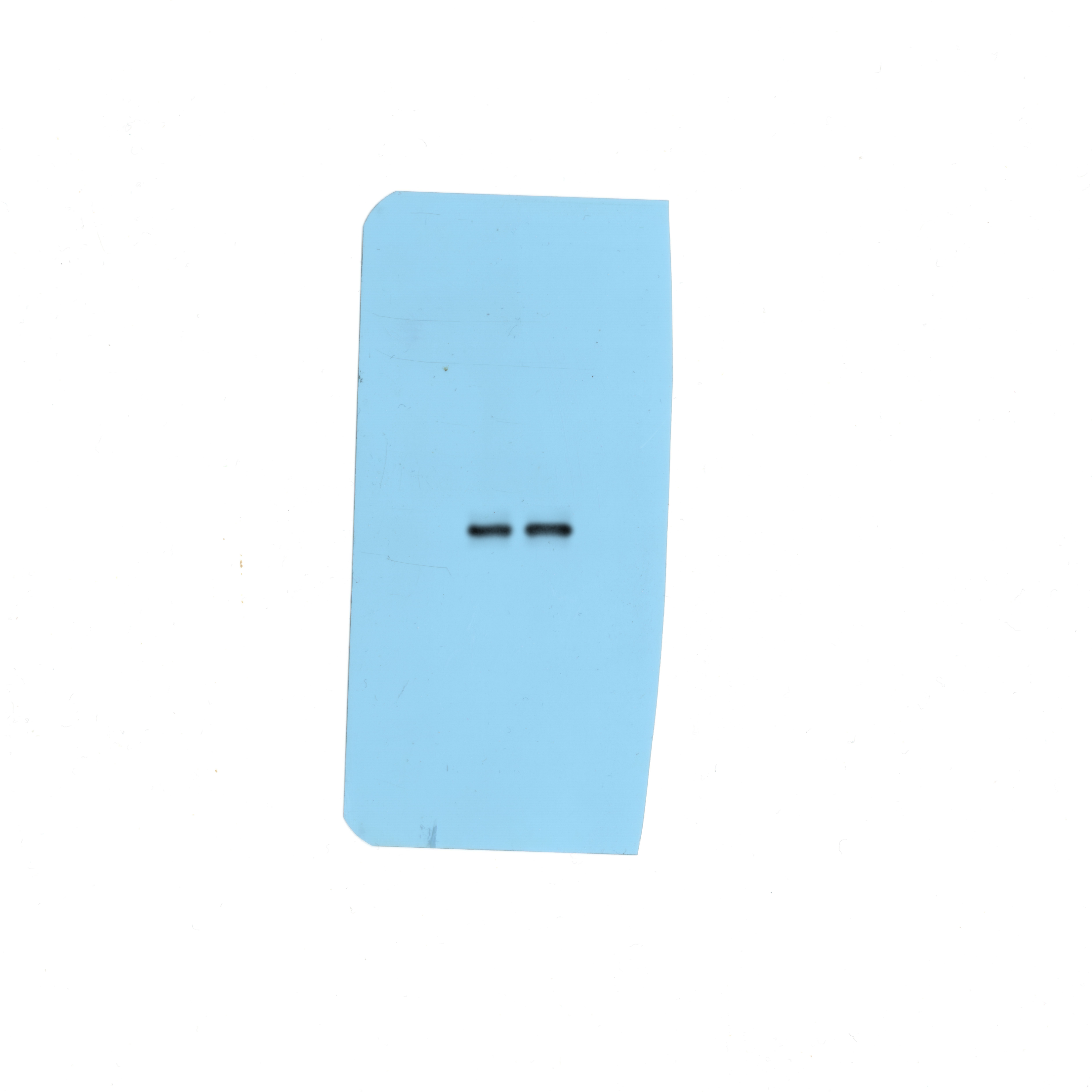

Supplement: Supplemental Information 8 [file peerj-12-16740-s008.zip › WB-Fugure 6/Figure 6B_GAPDH_CCLP1.tif]

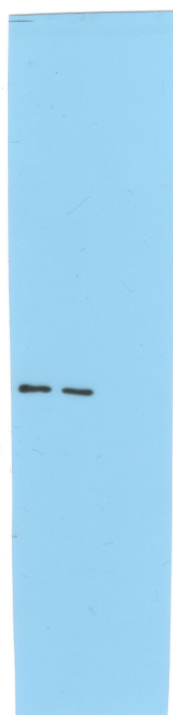

Supplement: Supplemental Information 8 [file peerj-12-16740-s008.zip › WB-Fugure 6/Figure 6B_GAPDH_HuCCT1.pdf]

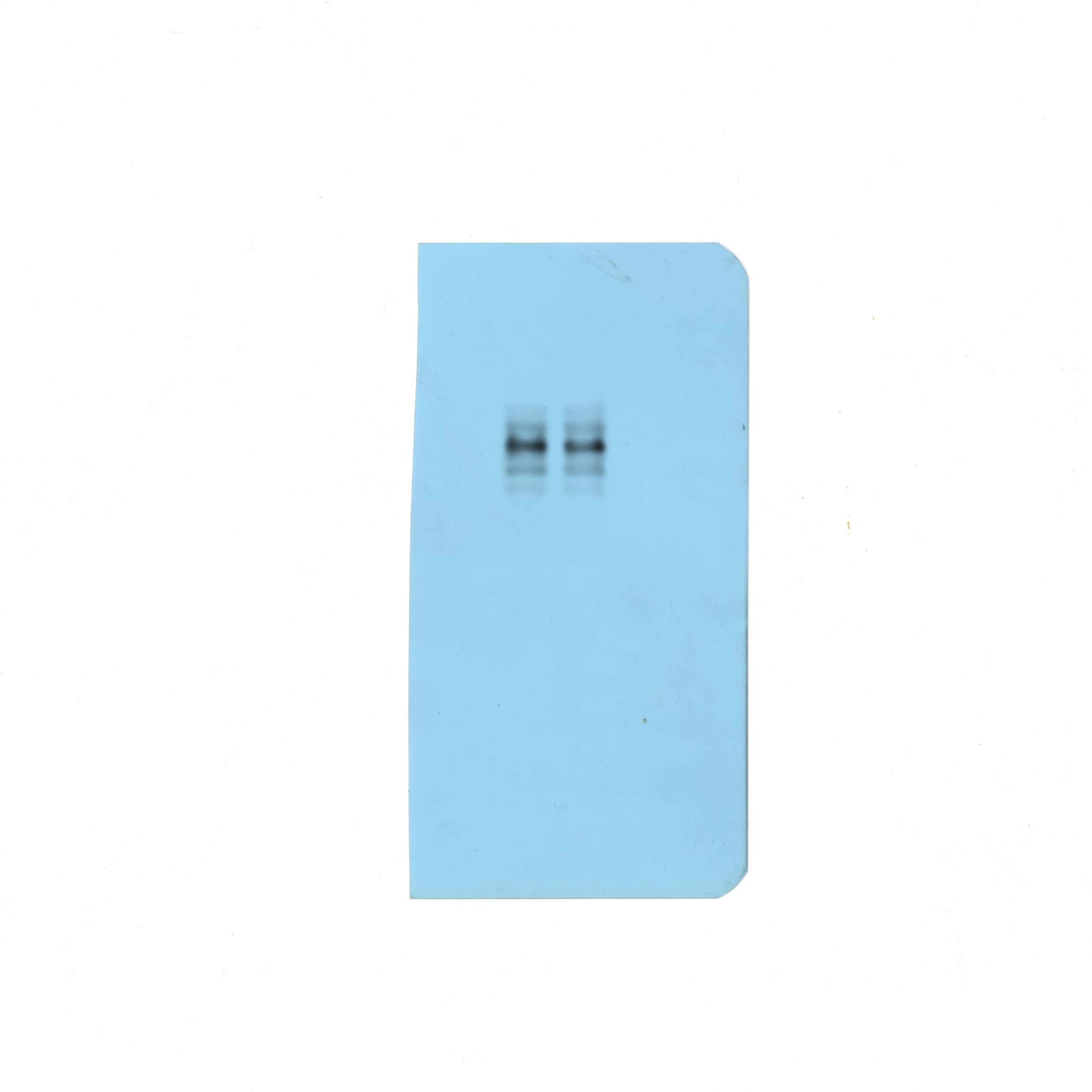

Supplement: Supplemental Information 8 [file peerj-12-16740-s008.zip › WB-Fugure 6/Figure 6C_ABCA1_cytosolic.tif]

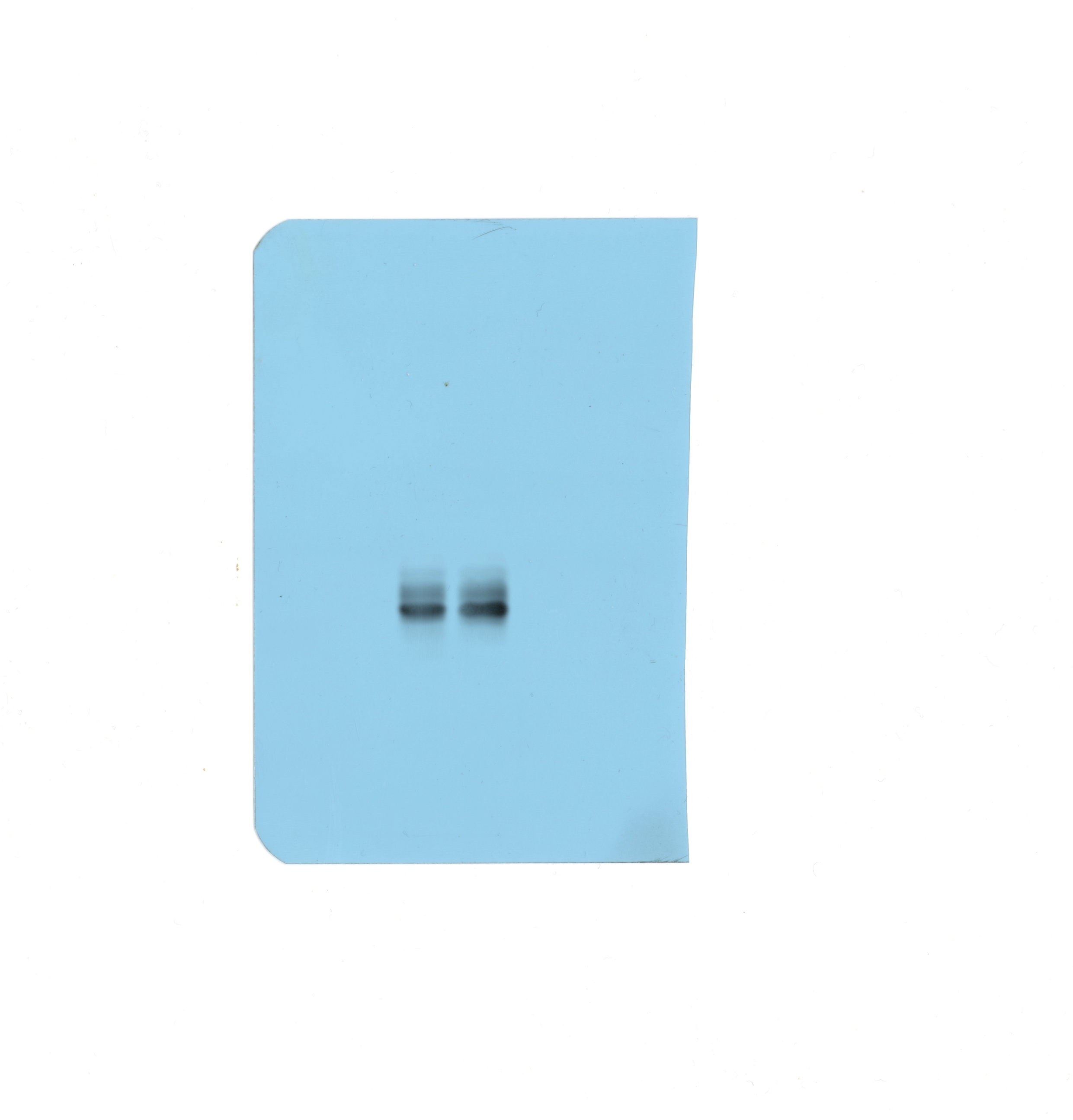

Supplement: Supplemental Information 8 [file peerj-12-16740-s008.zip › WB-Fugure 6/Figure 6C_ABCA1_MEM.tif]

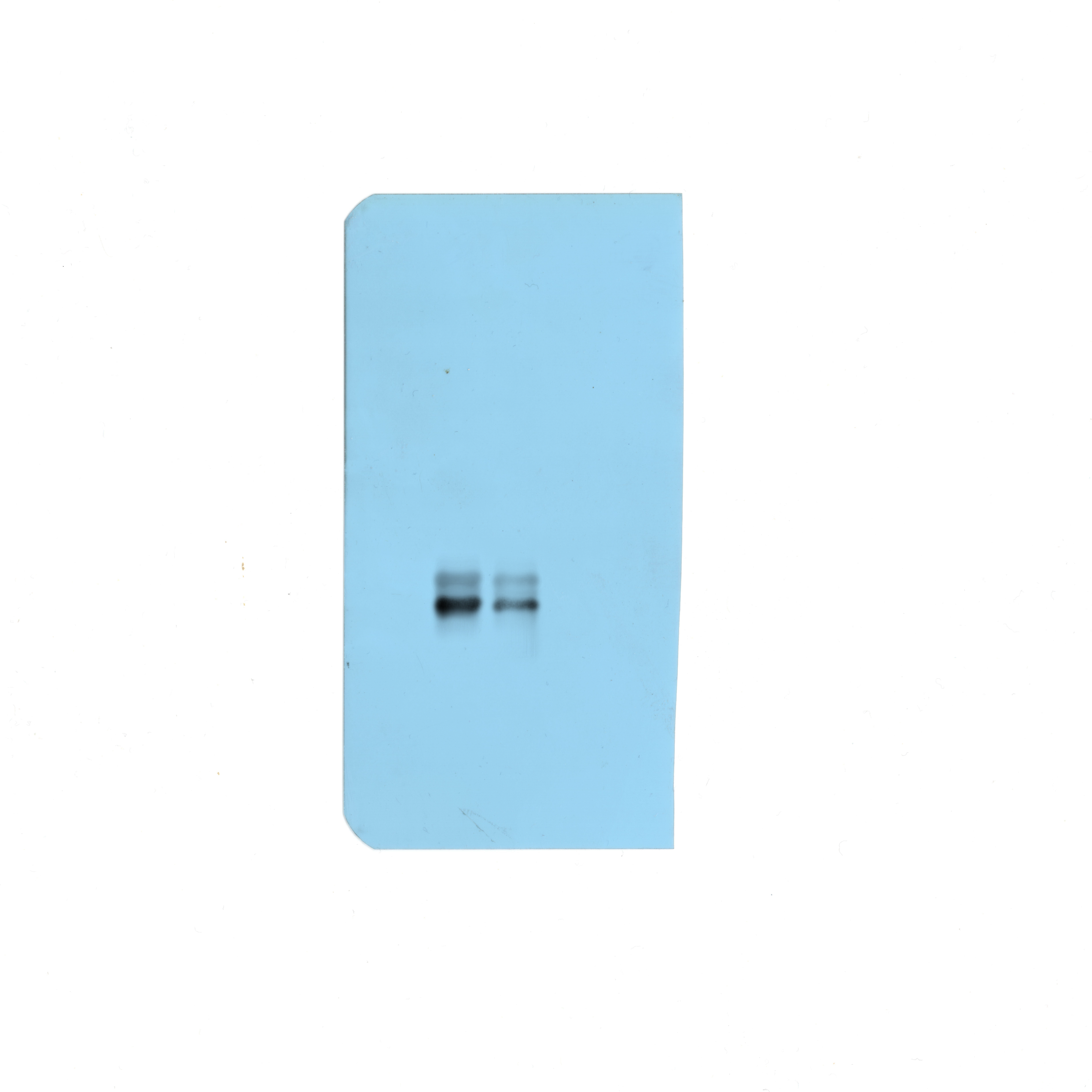

Supplement: Supplemental Information 8 [file peerj-12-16740-s008.zip › WB-Fugure 6/Figure 6C_ATPase_MEM.tif]

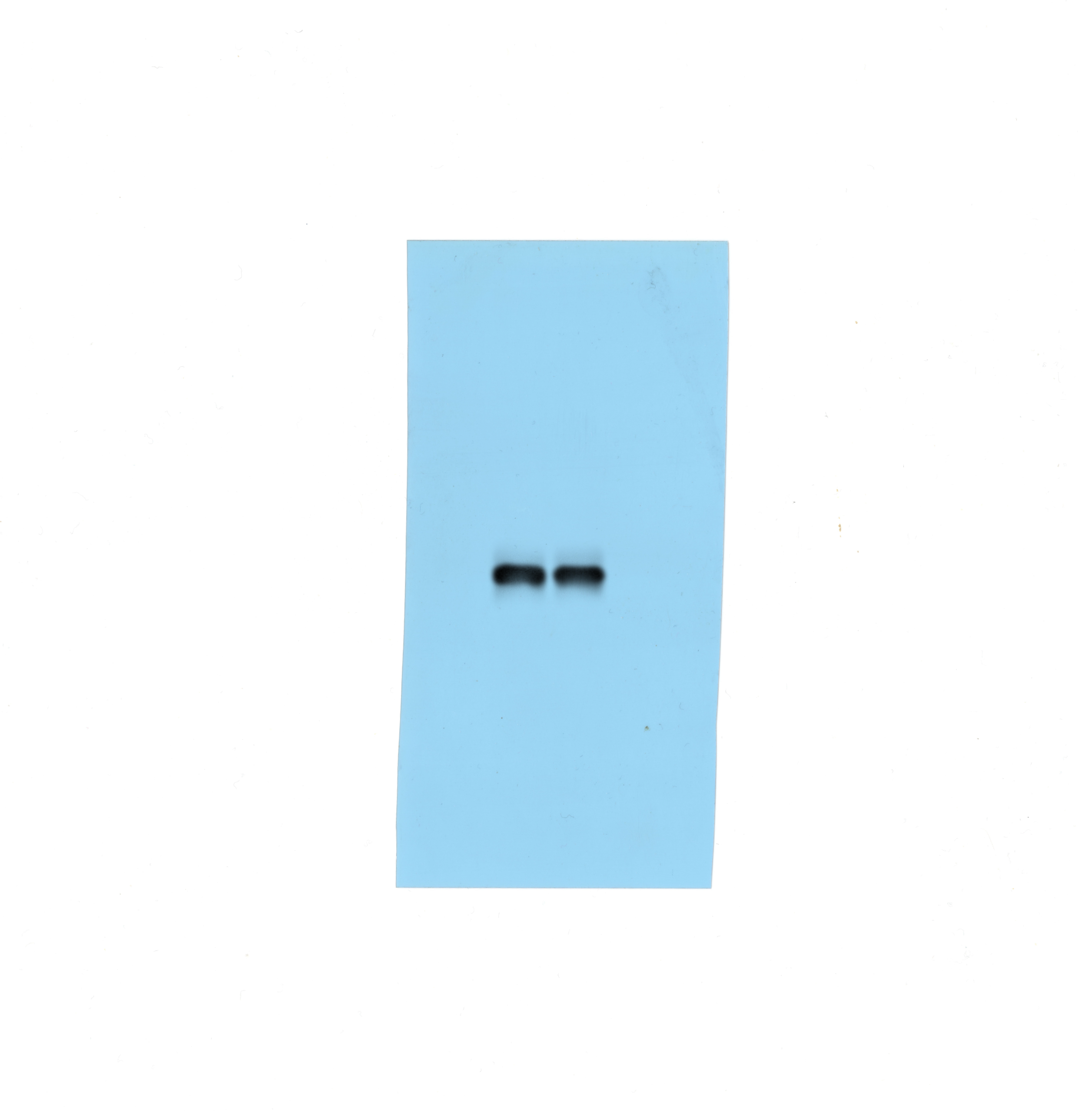

Supplement: Supplemental Information 8 [file peerj-12-16740-s008.zip › WB-Fugure 6/Figure 6C_GAPDH_cytosolic.tif]

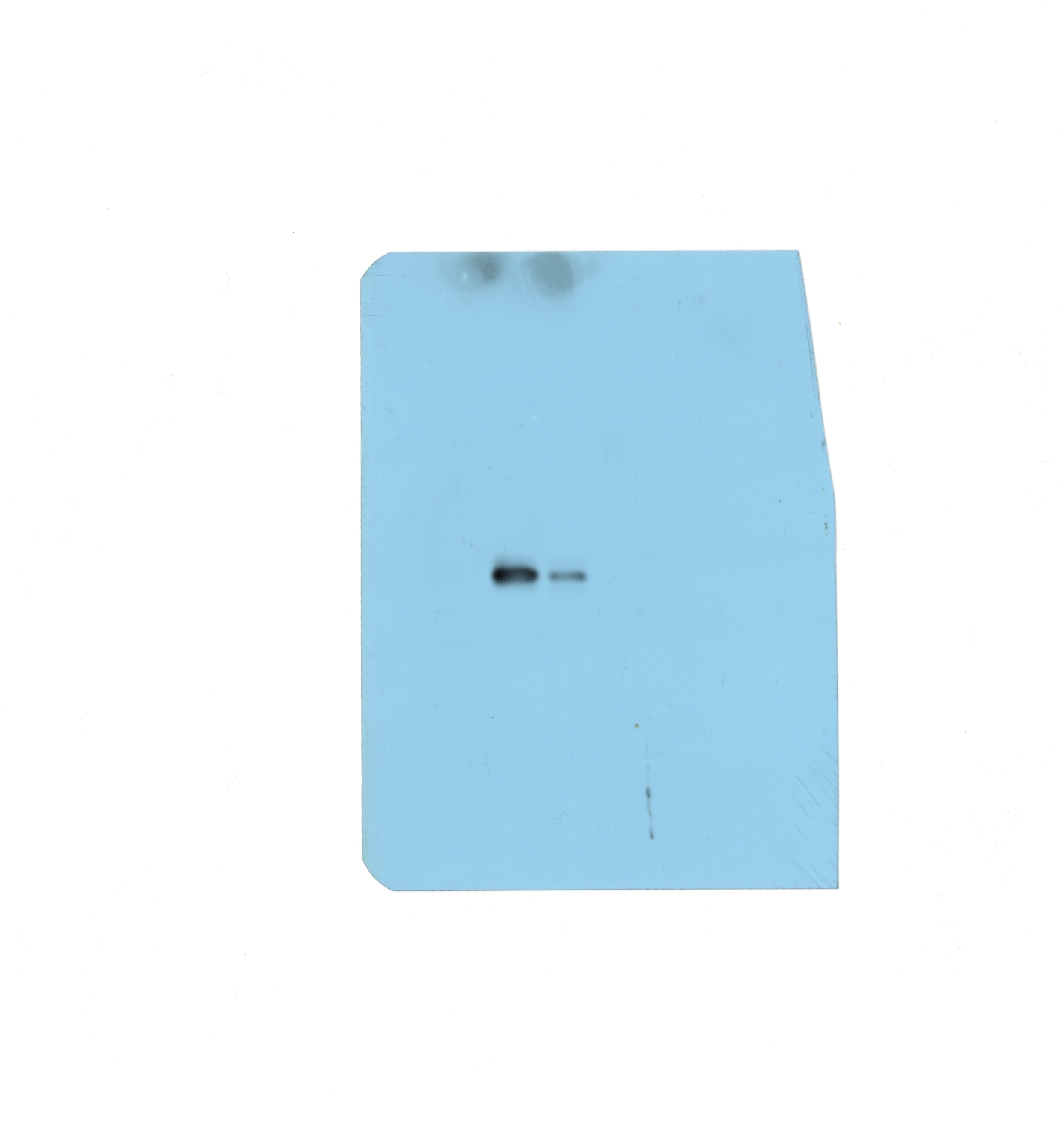

Supplement: Supplemental Information 8 [file peerj-12-16740-s008.zip › WB-Fugure 6/Figure 6E_ABCA1_CCLP1.tif]

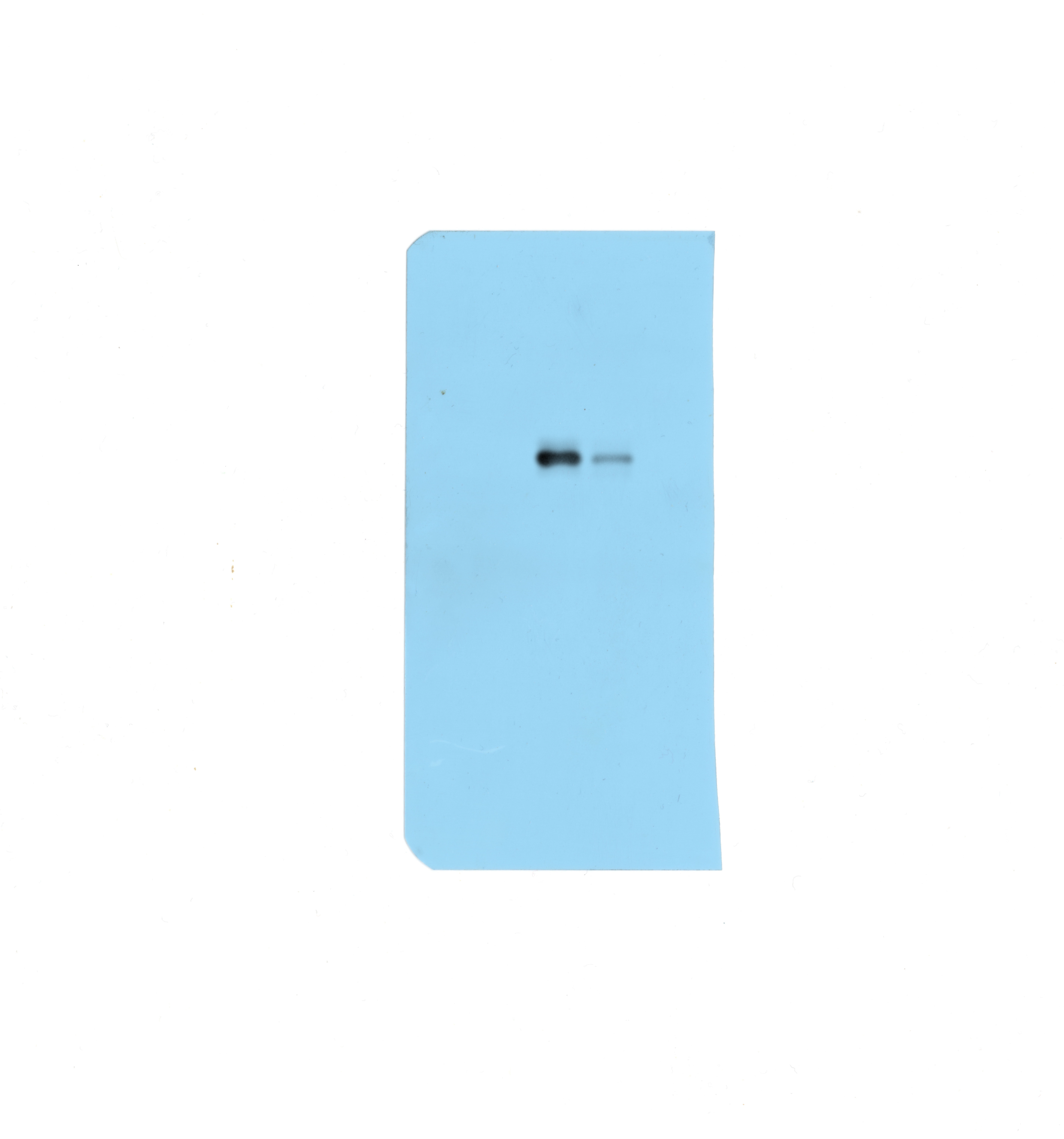

Supplement: Supplemental Information 8 [file peerj-12-16740-s008.zip › WB-Fugure 6/Figure 6E_ABCA1_HuCCT1.tif]

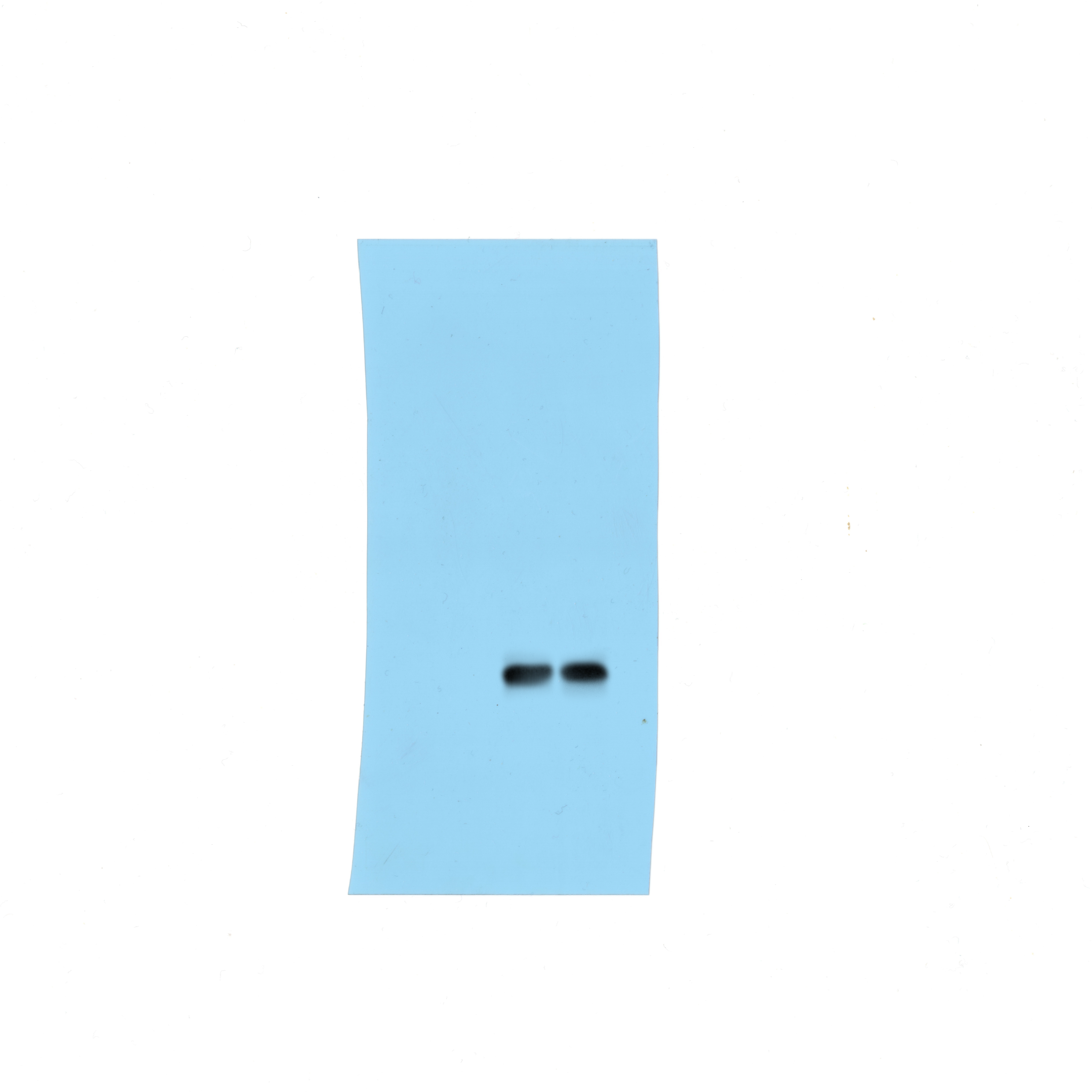

Supplement: Supplemental Information 8 [file peerj-12-16740-s008.zip › WB-Fugure 6/Figure 6E_GAPDH_CCLP1.tif]

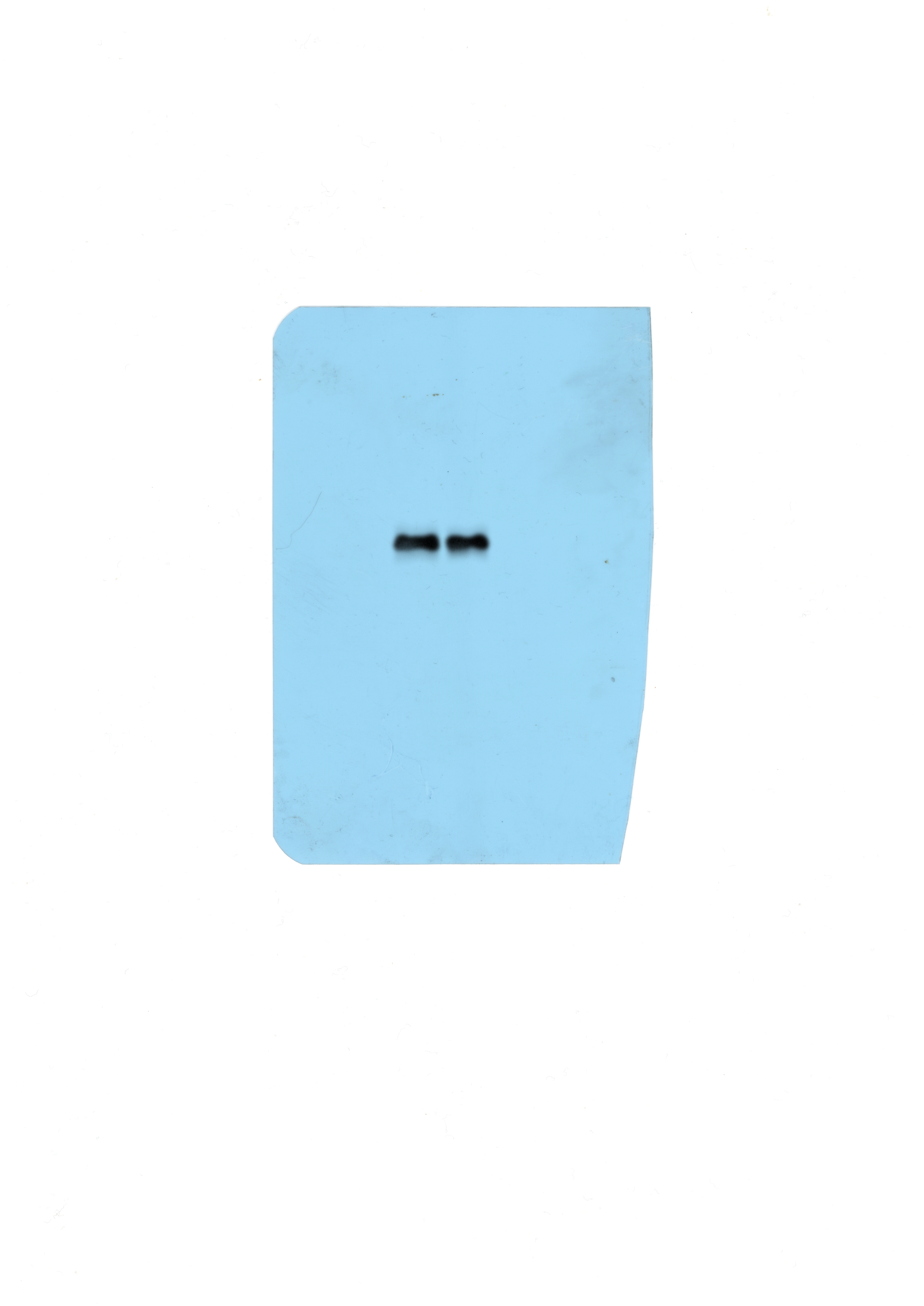

Supplement: Supplemental Information 8 [file peerj-12-16740-s008.zip › WB-Fugure 6/Figure 6E_GAPDH_HuCCT1.tif]

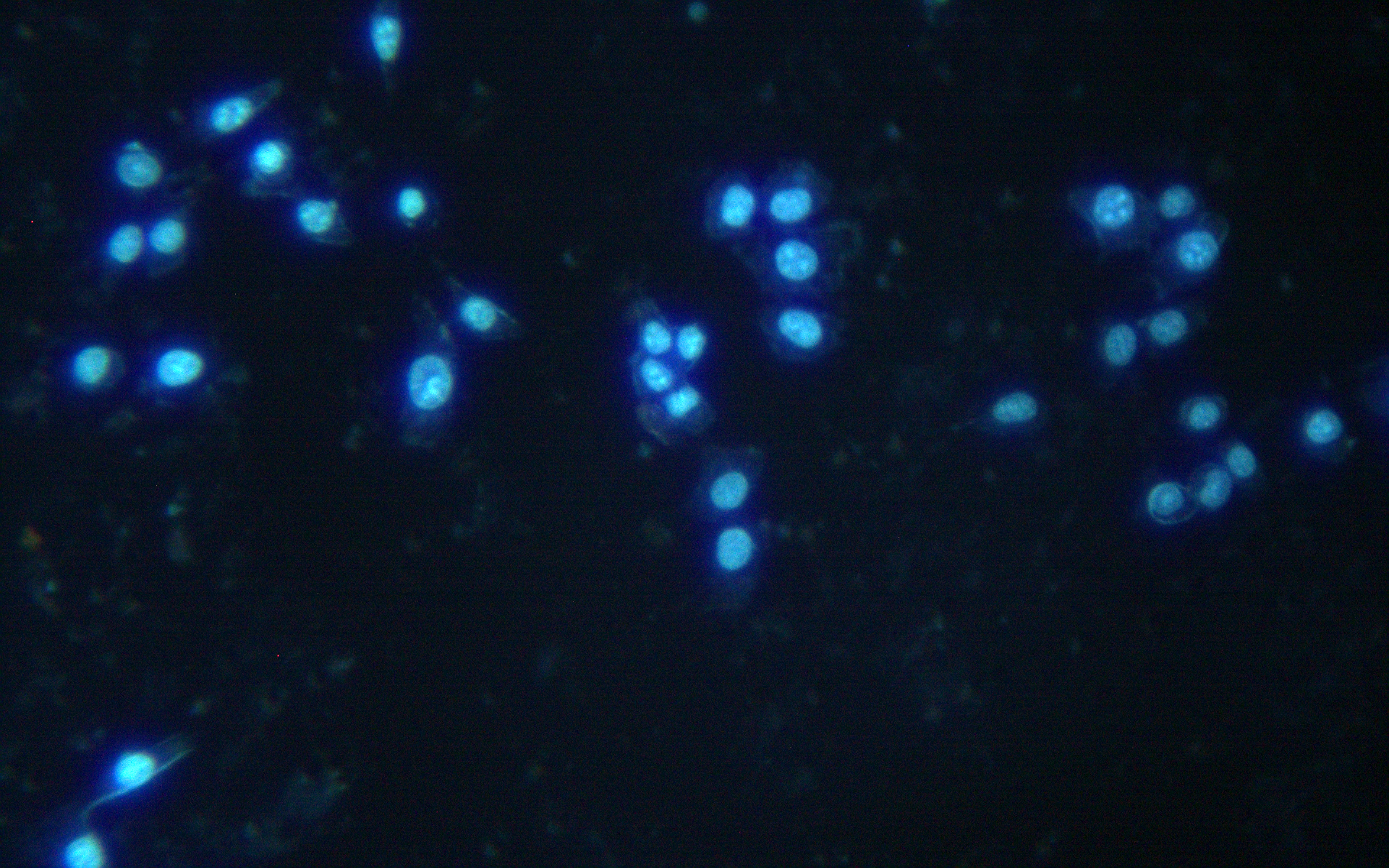

Supplement: Supplemental Information 9 [file peerj-12-16740-s009.zip › transwell-migration-CCLP1/Figure 4F_over-APOE4_CCLP1_Migration.tif]

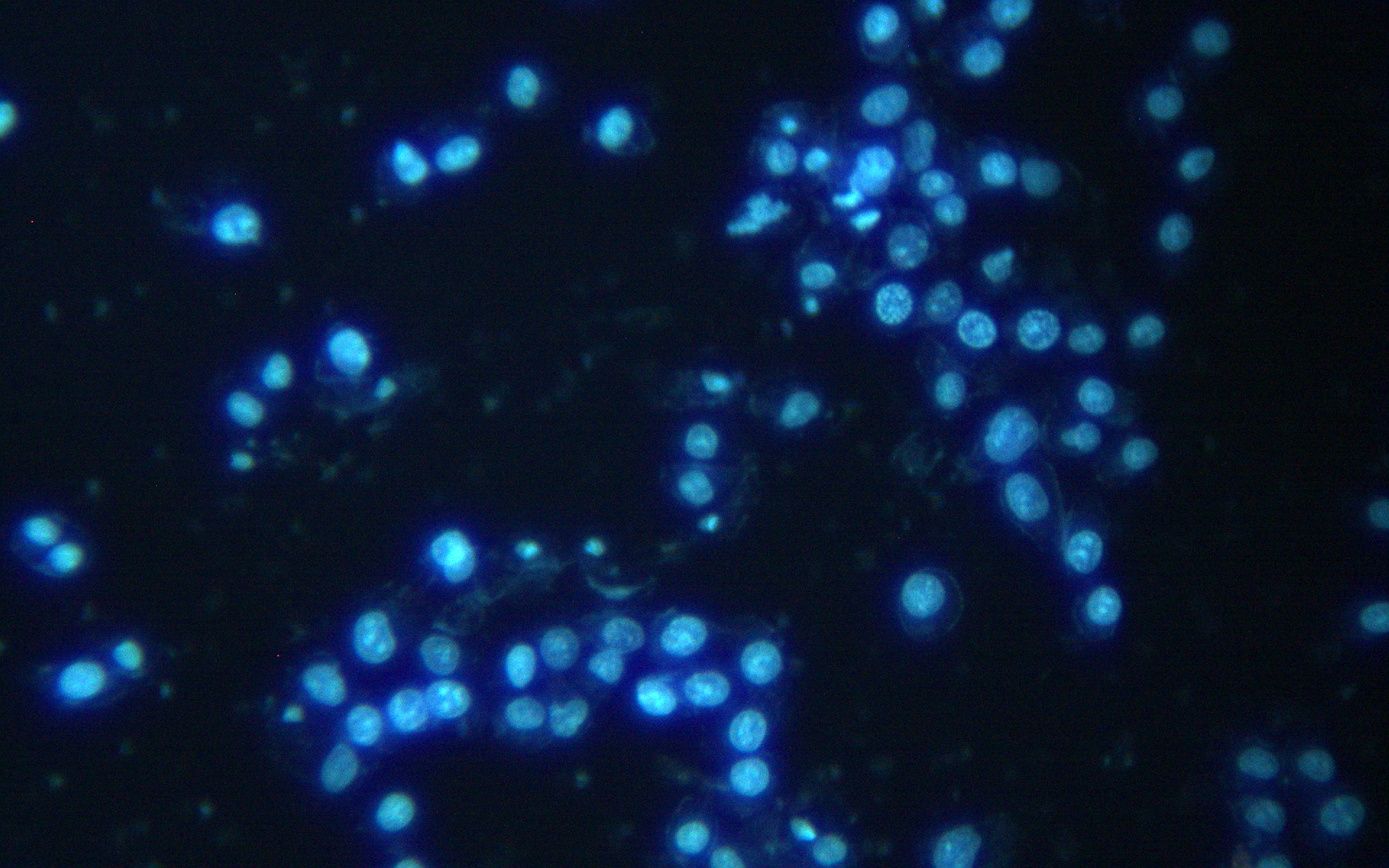

Supplement: Supplemental Information 9 [file peerj-12-16740-s009.zip › transwell-migration-CCLP1/Figure 4F_over-NC_CCLP1_Migration.tif]

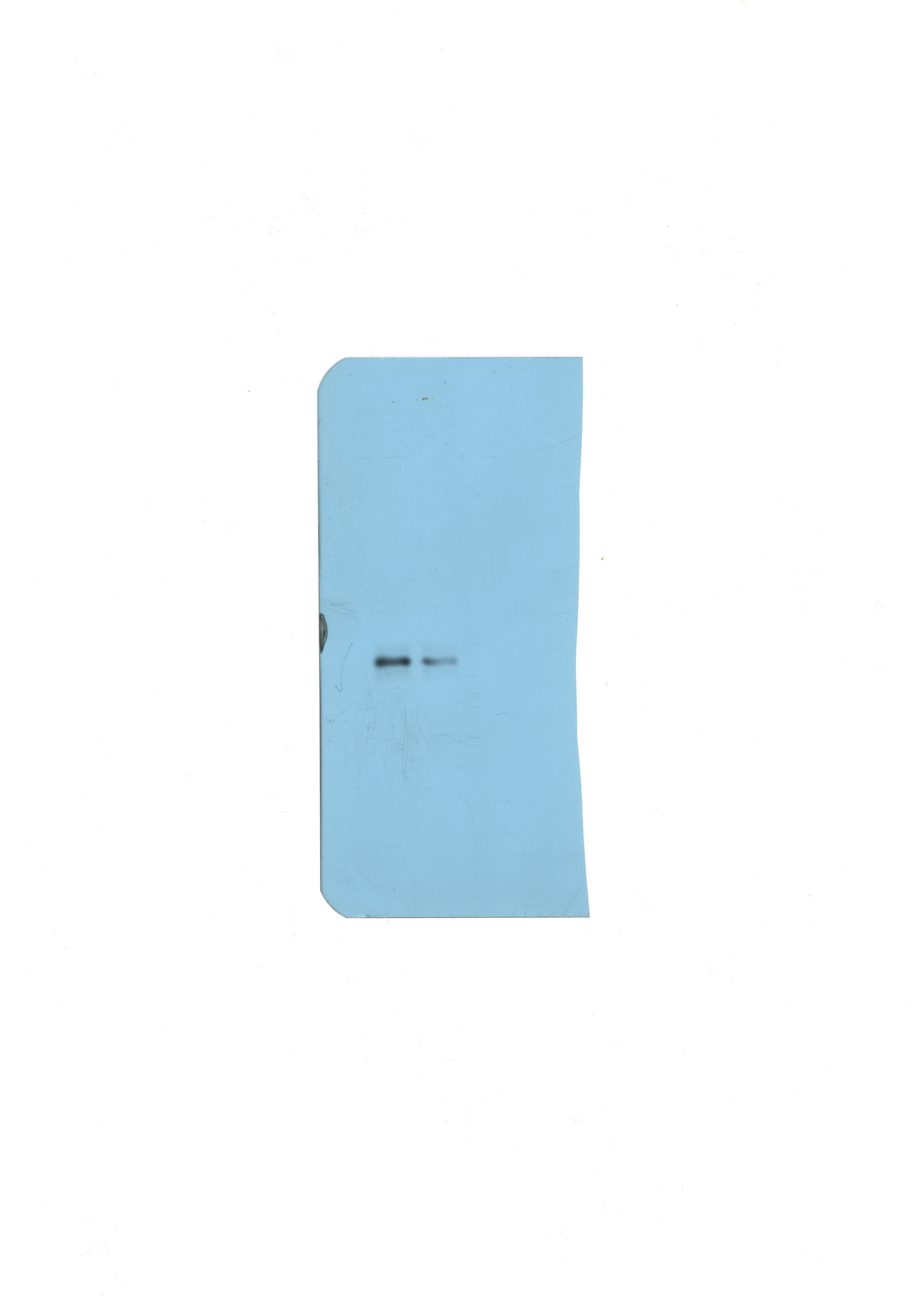

Supplement: Supplemental Information 10 [file peerj-12-16740-s010.zip › Figure 4C-HuCCT1/Figure 4C_ACC_HuCCT1.tif]

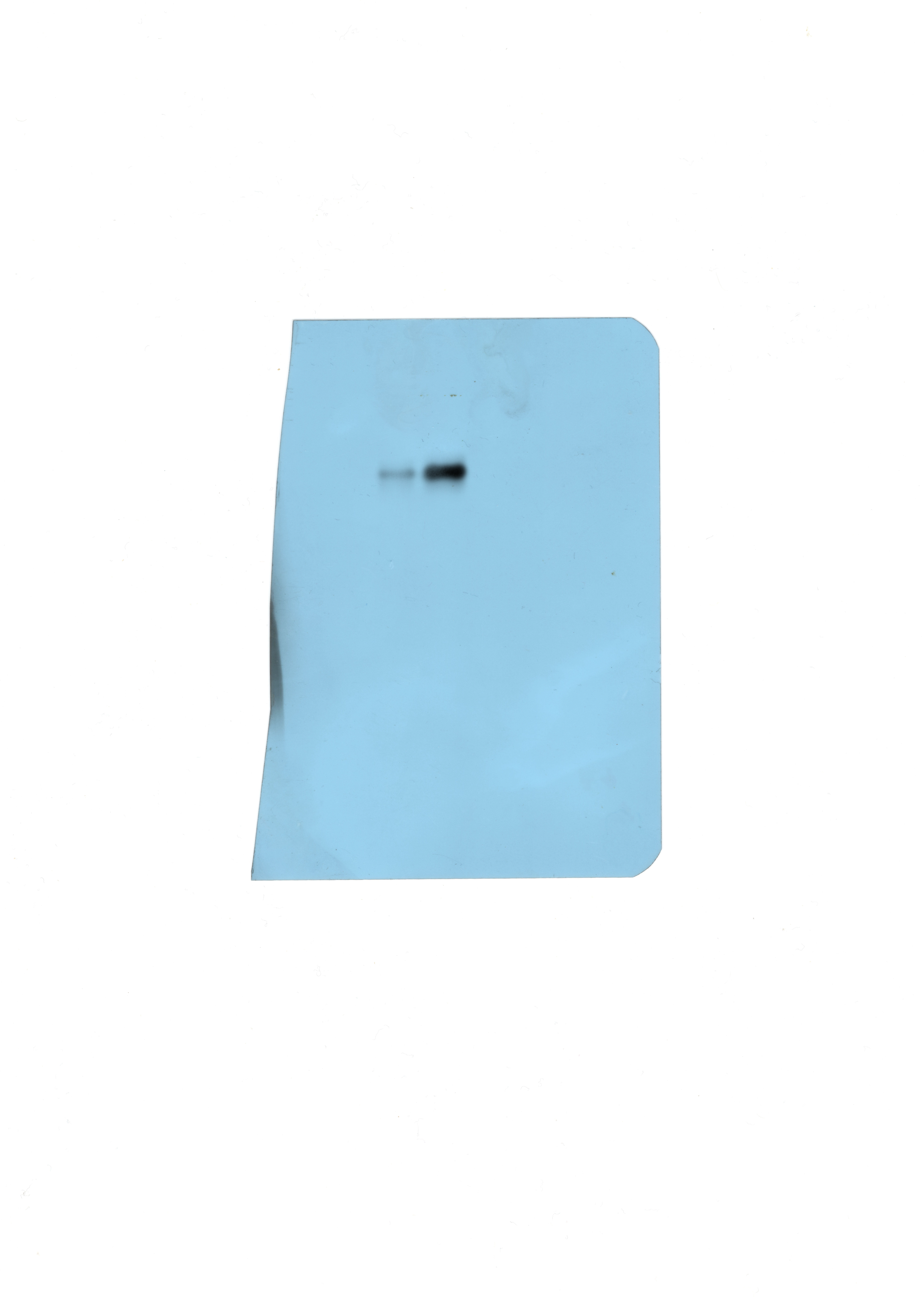

Supplement: Supplemental Information 10 [file peerj-12-16740-s010.zip › Figure 4C-HuCCT1/Figure 4C_APOE4_HuCCT1.tif]

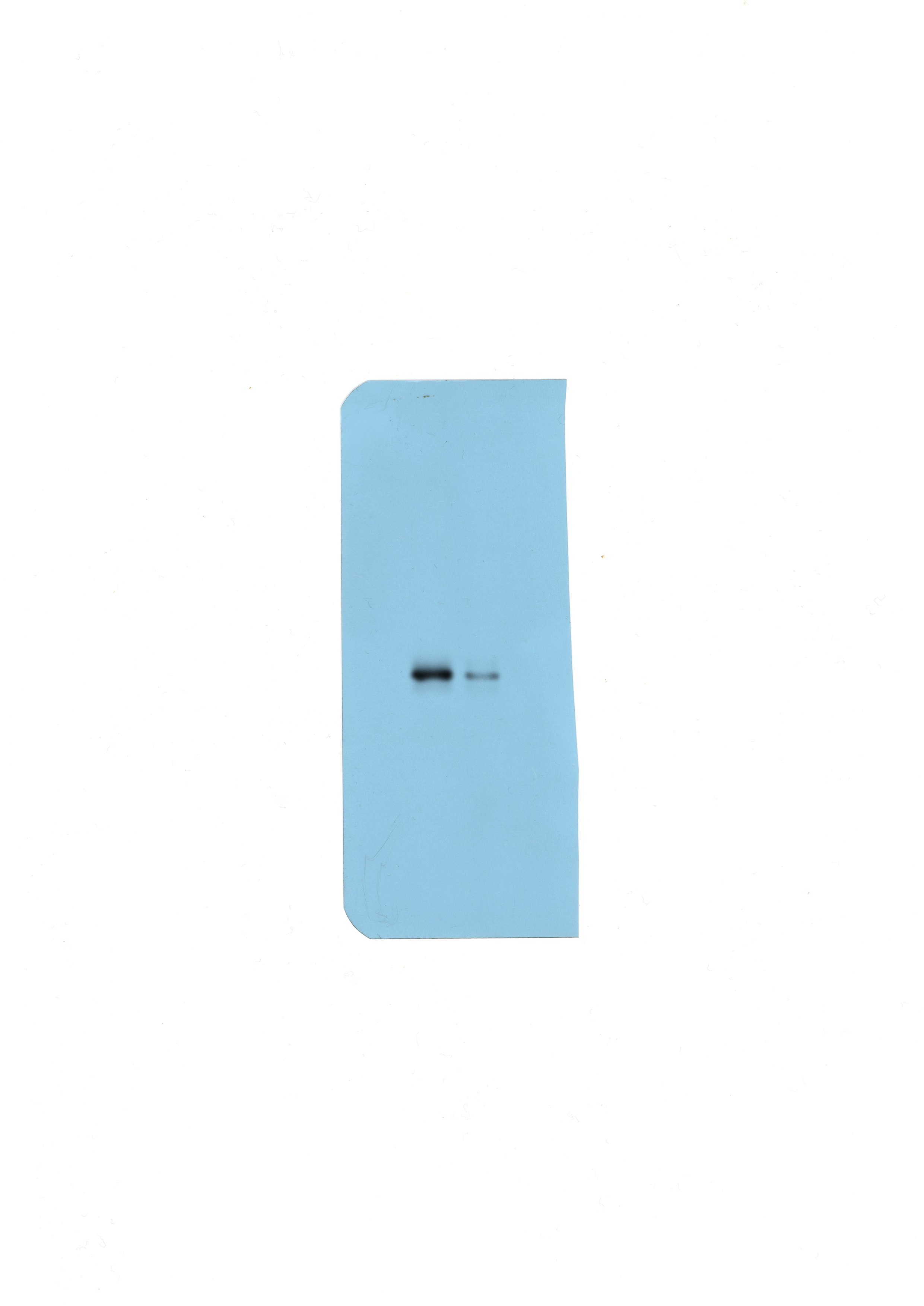

Supplement: Supplemental Information 10 [file peerj-12-16740-s010.zip › Figure 4C-HuCCT1/Figure 4C_FAS_HuCCT1.tif]

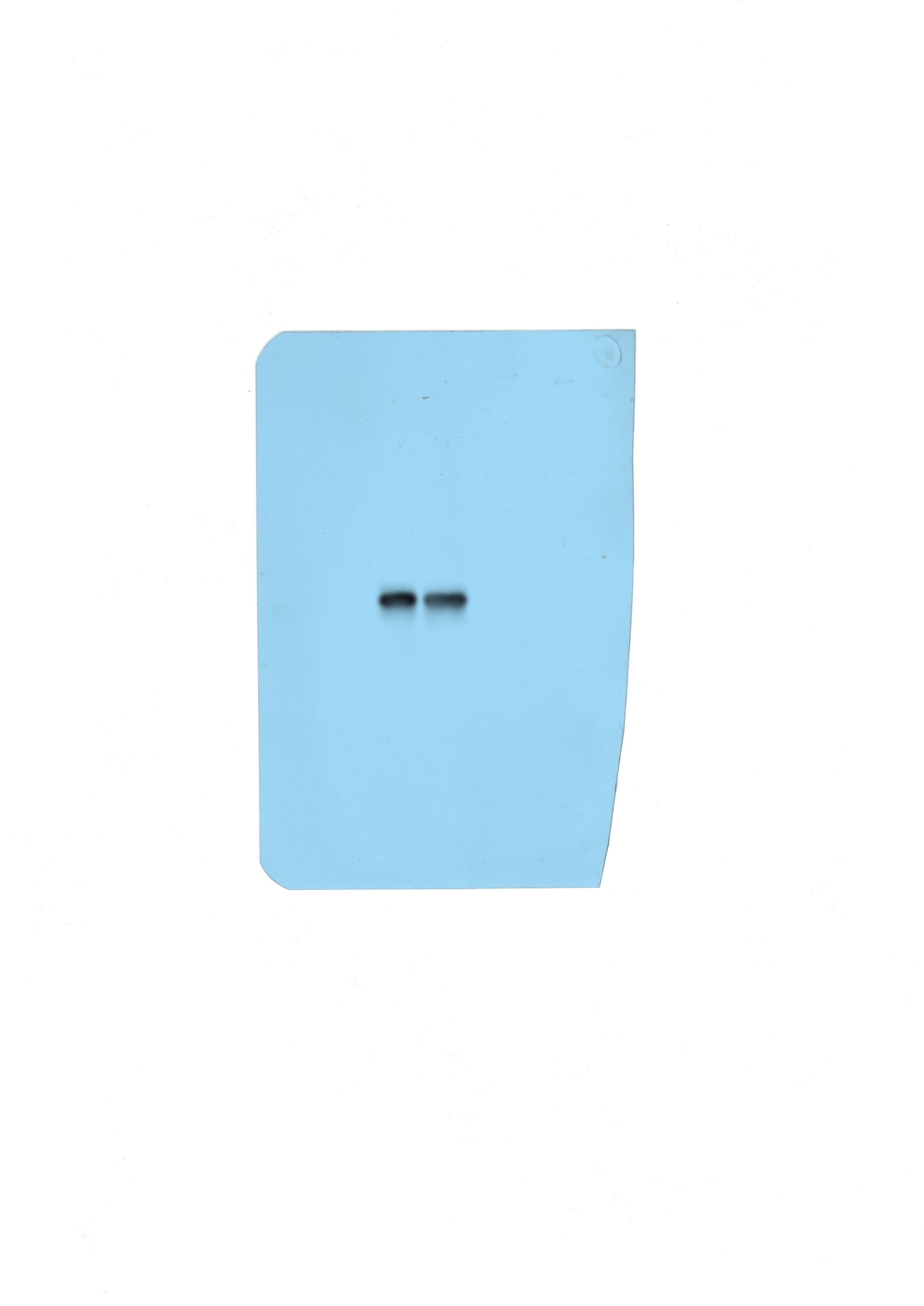

Supplement: Supplemental Information 10 [file peerj-12-16740-s010.zip › Figure 4C-HuCCT1/Figure 4C_GAPDH_HuCCT1.tif]

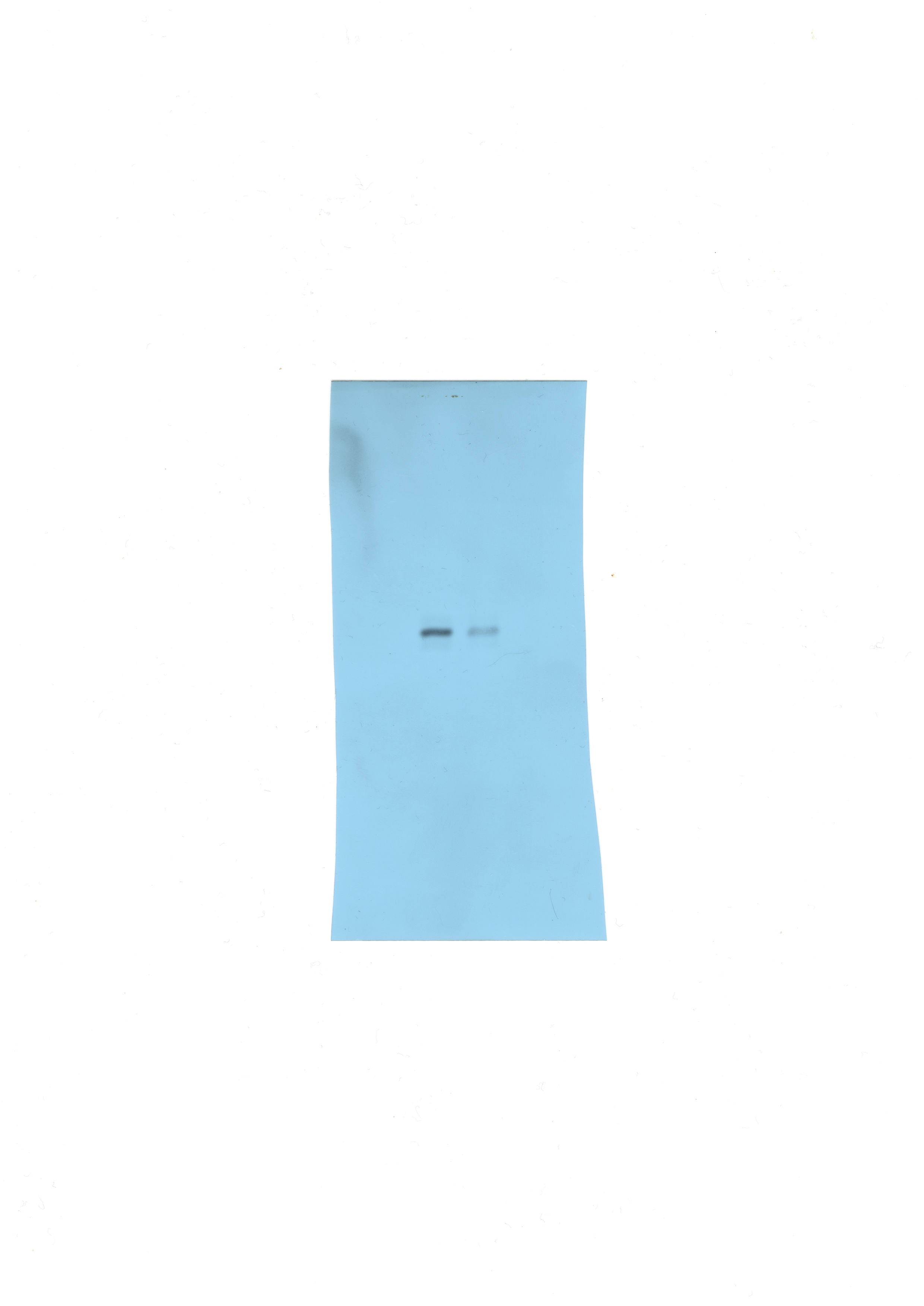

Supplement: Supplemental Information 10 [file peerj-12-16740-s010.zip › Figure 4C-HuCCT1/Figure 4C_GPT-1_HuCCT1.tif]

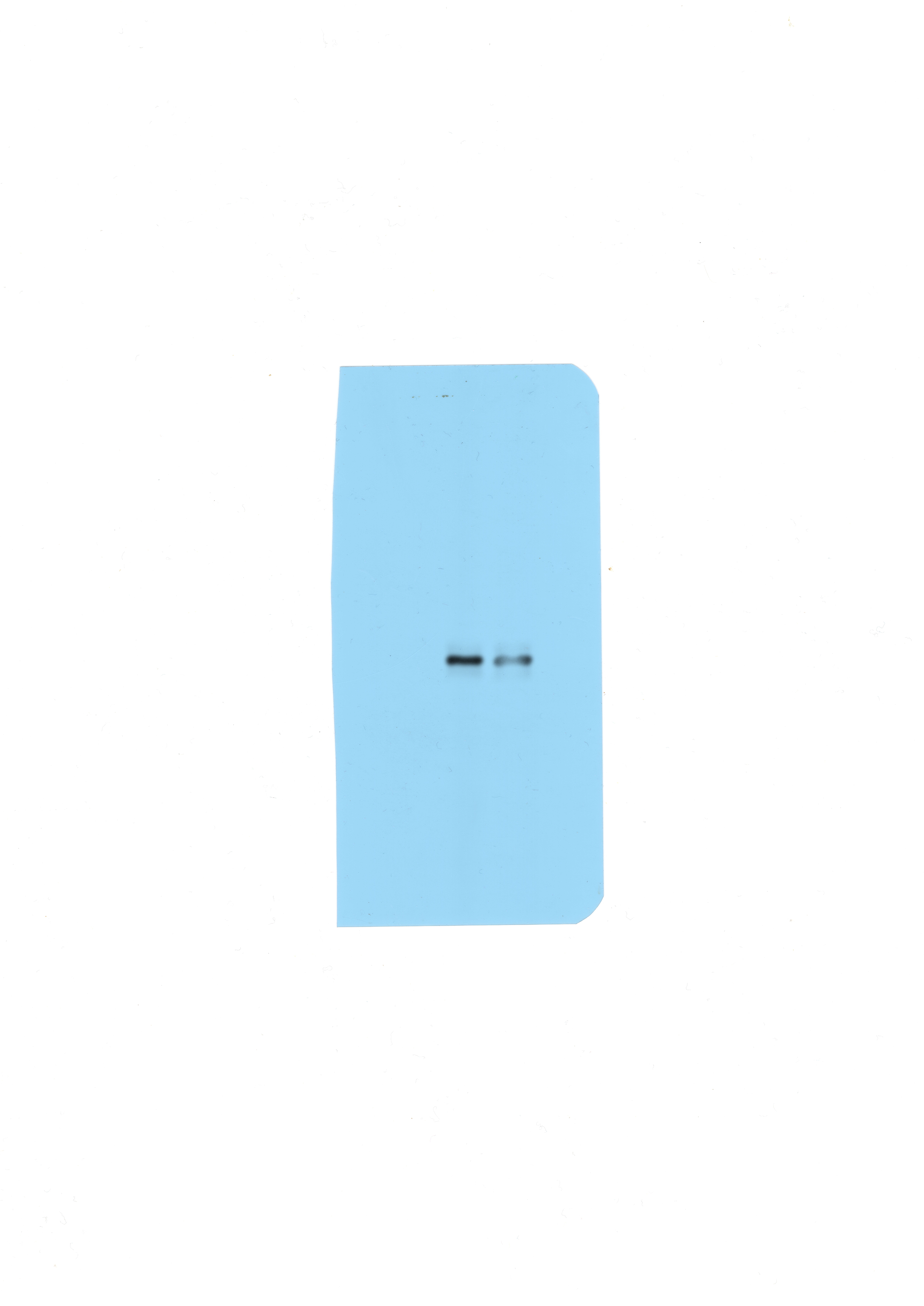

Supplement: Supplemental Information 10 [file peerj-12-16740-s010.zip › Figure 4C-HuCCT1/Figure 4C_SCD1_HuCCT1.tif]

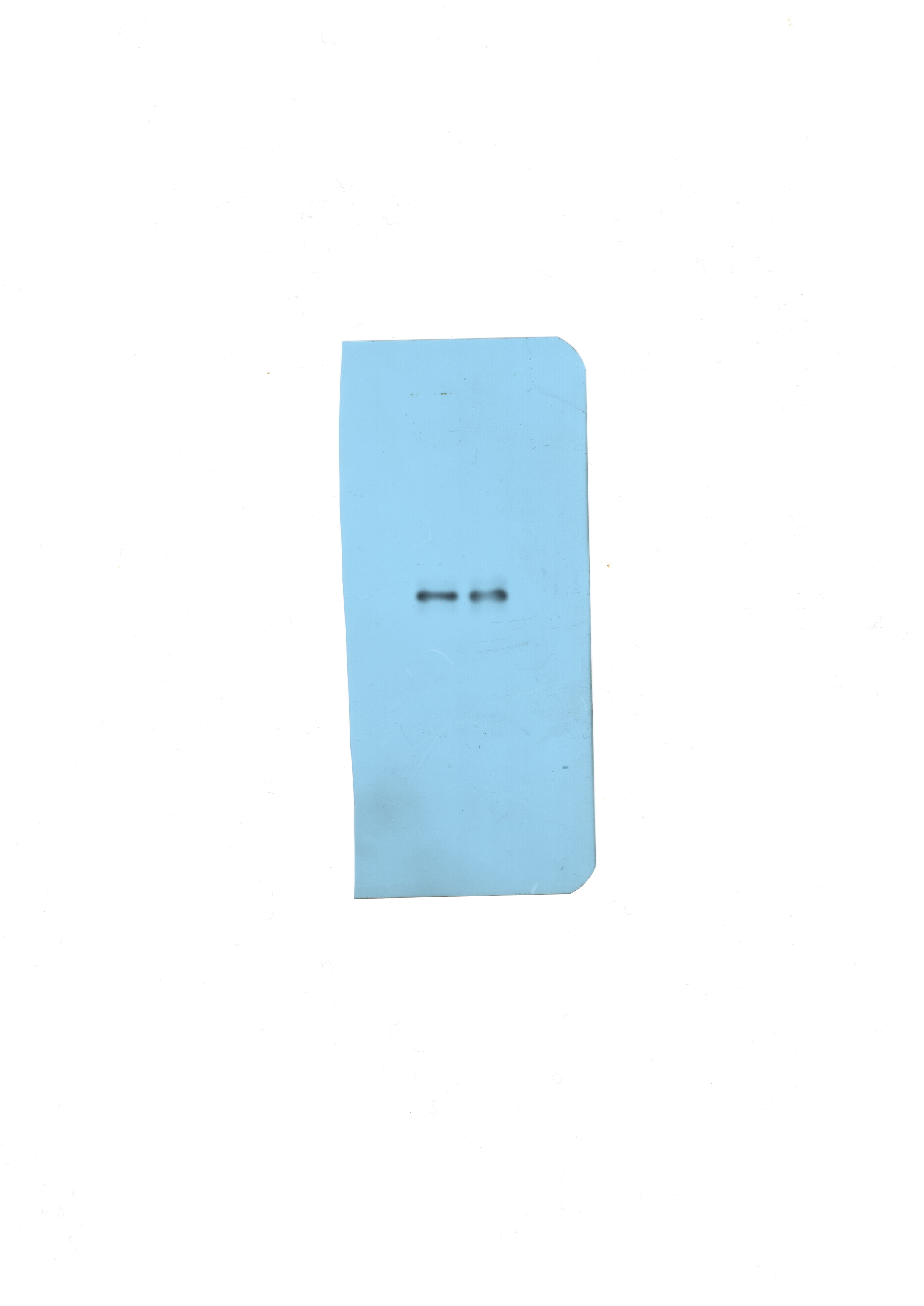

Supplement: Supplemental Information 10 [file peerj-12-16740-s010.zip › Figure 4C-HuCCT1/Figure 4C_SREBP1_HuCCT1.tif]

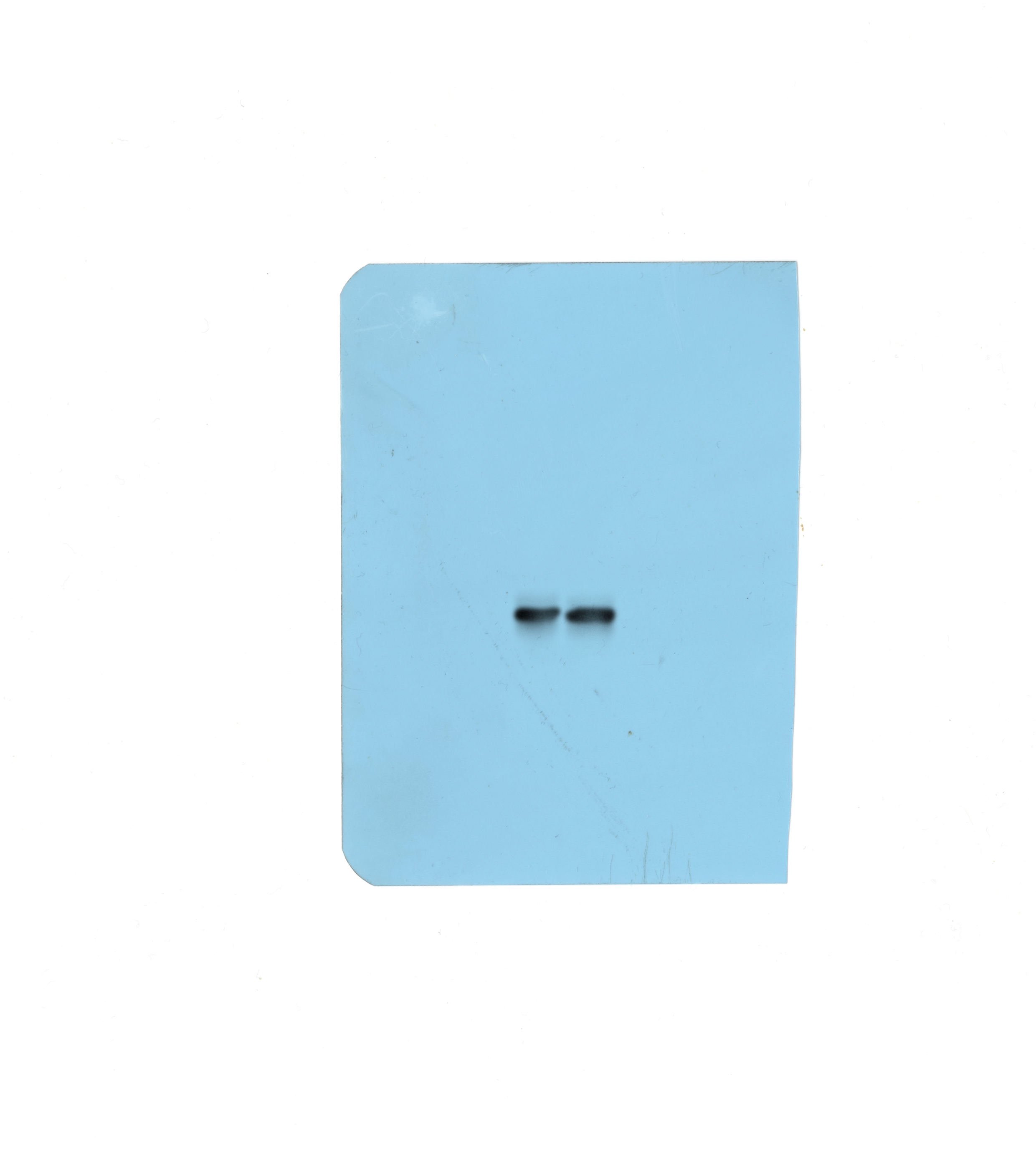

Supplement: Supplemental Information 11 [file peerj-12-16740-s011.zip › WB Figure 5-HuCCT1/Figure 5M_GAPDH_HuCCT1.tif]

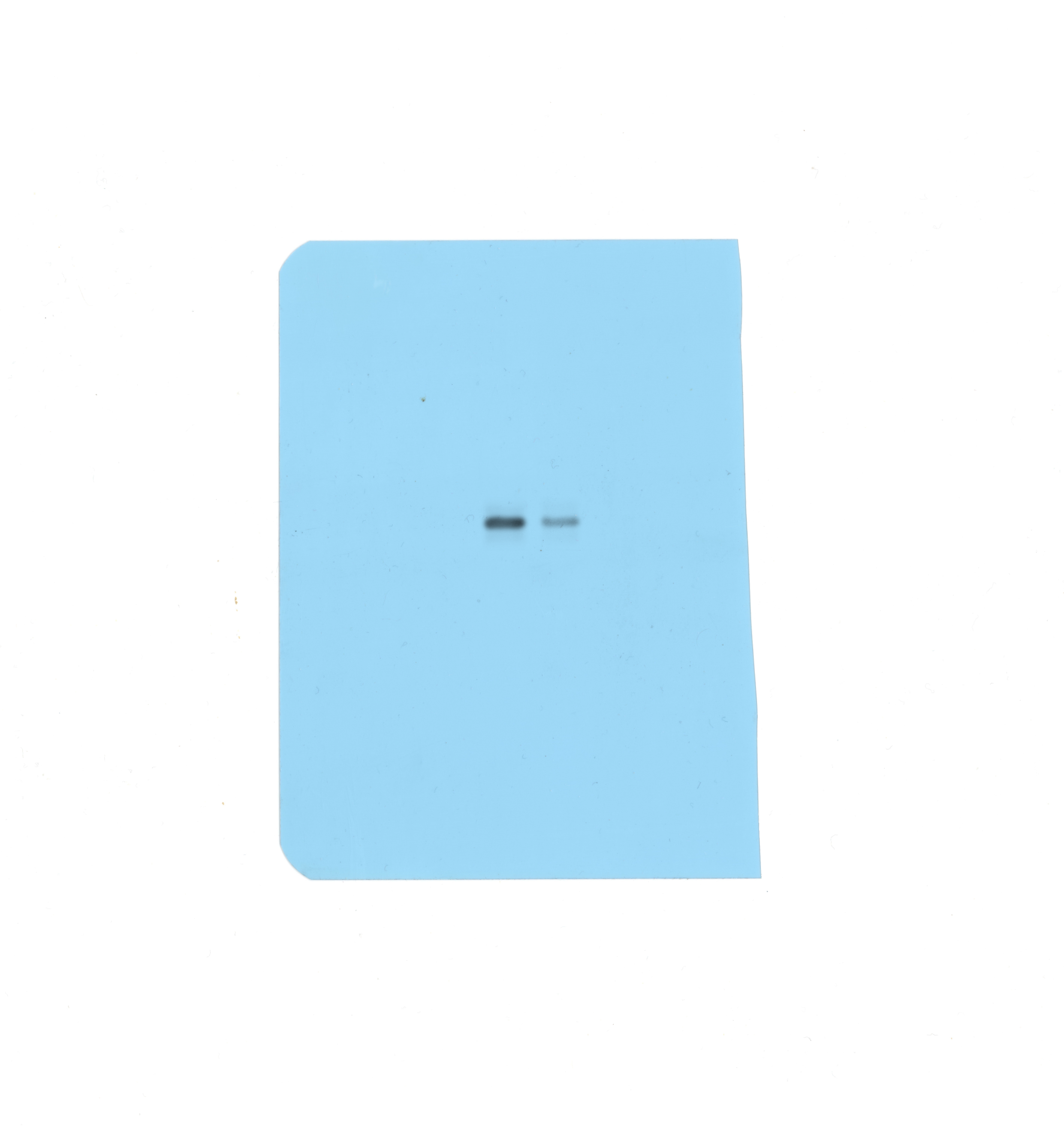

Supplement: Supplemental Information 11 [file peerj-12-16740-s011.zip › WB Figure 5-HuCCT1/Figure 5M_PPARa├_HuCCT1.tif]

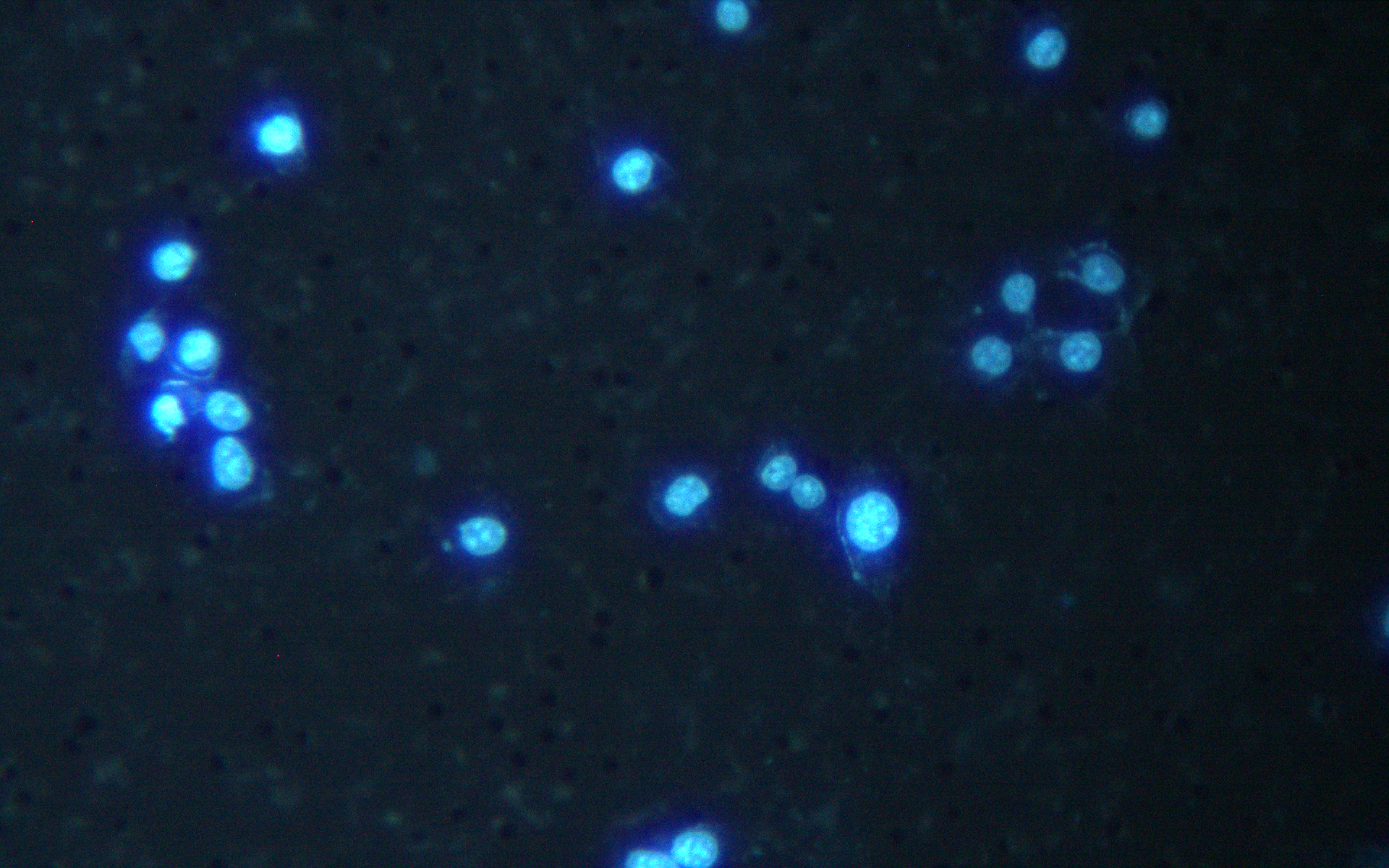

Supplement: Supplemental Information 12 [file peerj-12-16740-s012.zip › transwell-invasion-HuCCT1/Figure 4H_over-APOE4_HuCCT1_Invasion.tif]

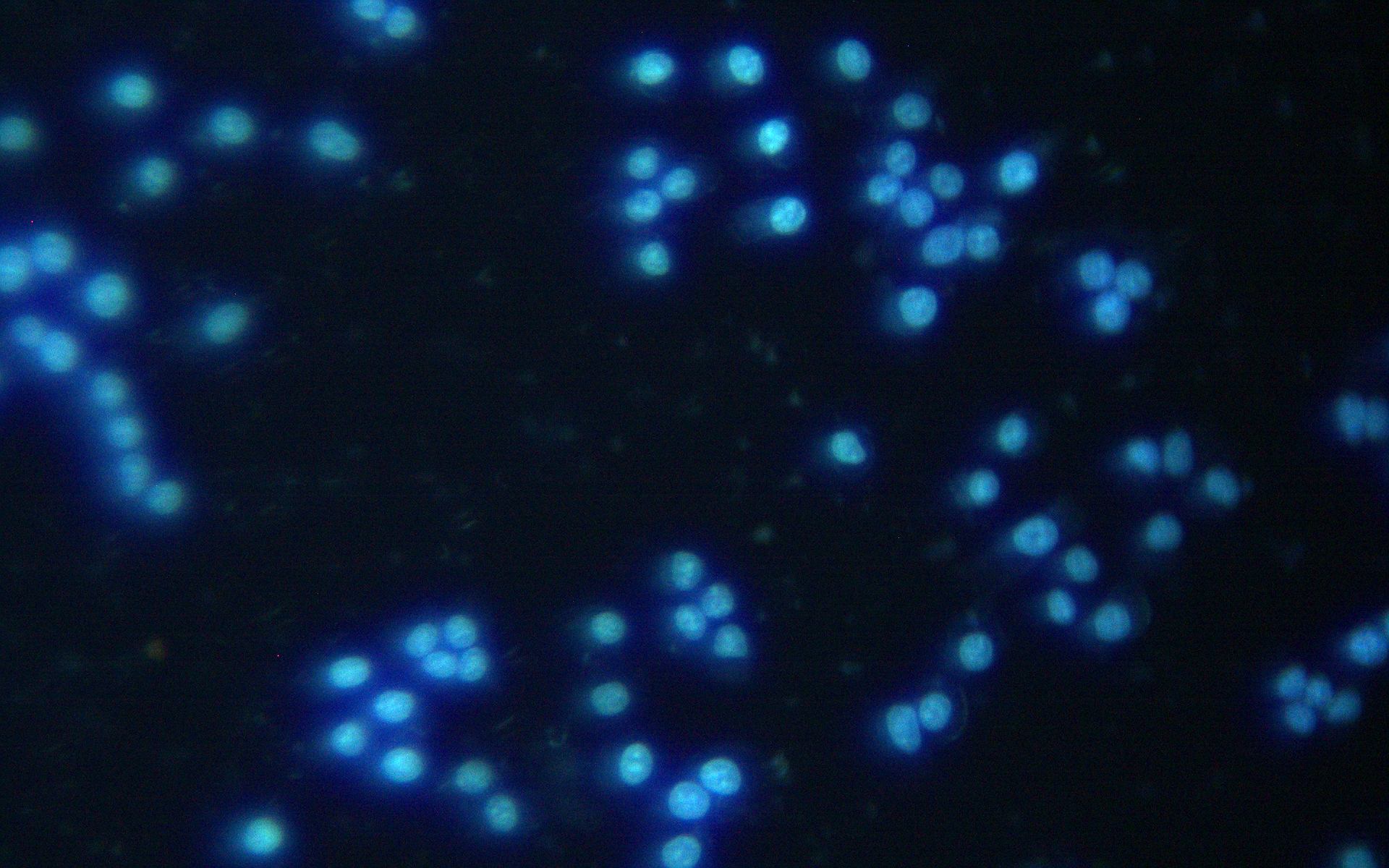

Supplement: Supplemental Information 12 [file peerj-12-16740-s012.zip › transwell-invasion-HuCCT1/Figure 4H_over-NC_HuCCT1_Invasion.tif]

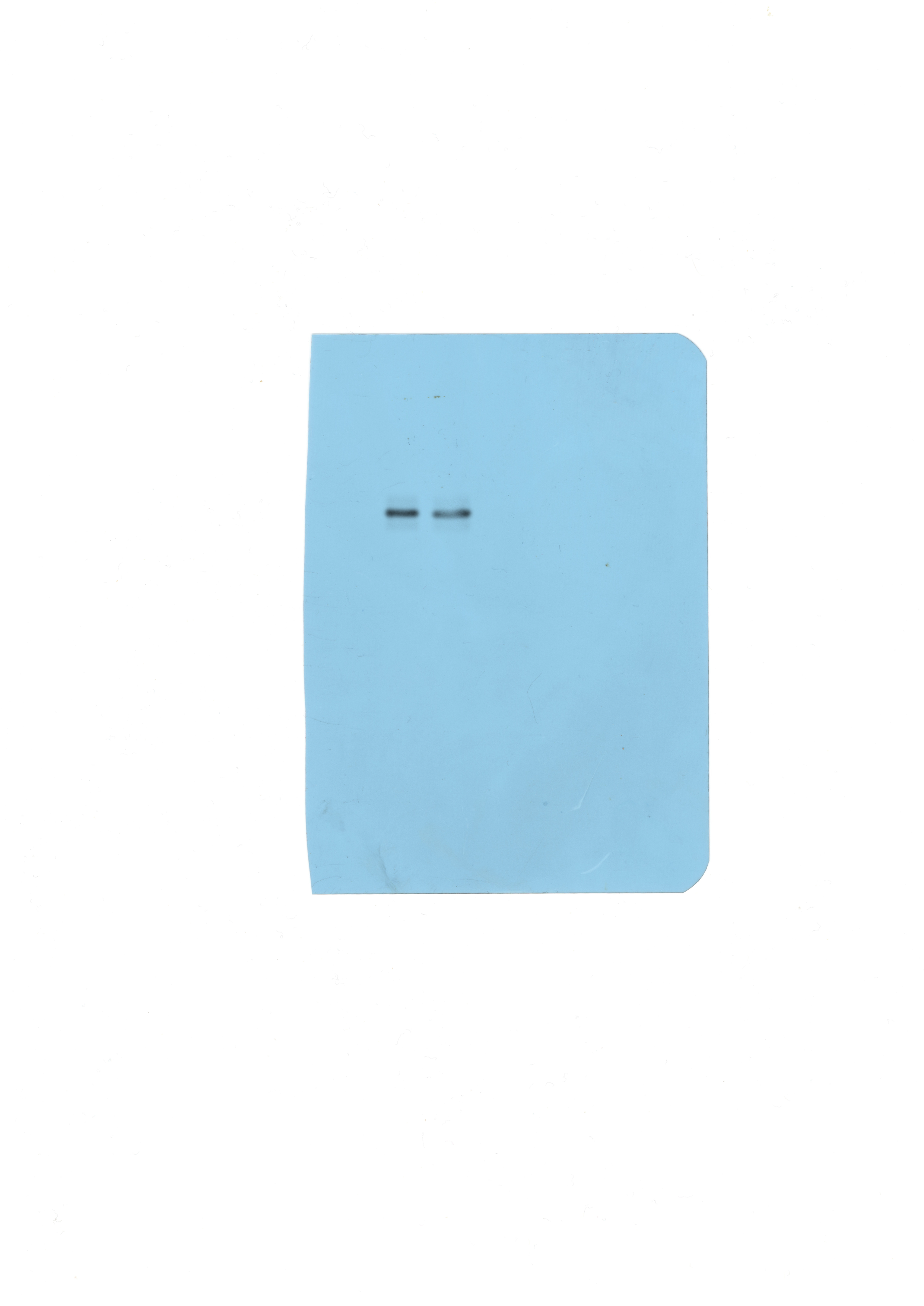

Supplement: Supplemental Information 13 [file peerj-12-16740-s013.zip › WB-Figure 5_CCLP1/Figure 5M_ACC_CCLP1.tif]

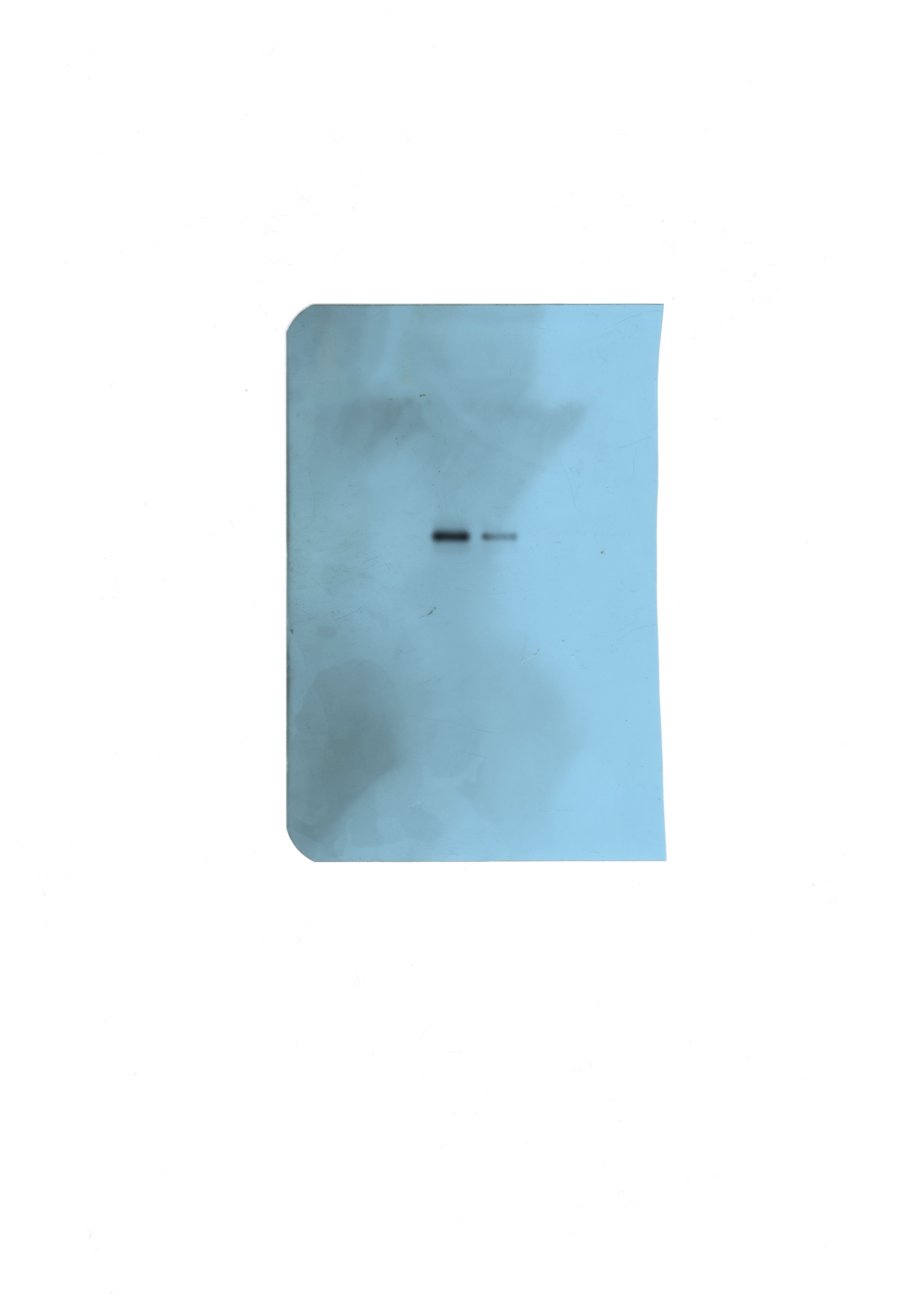

Supplement: Supplemental Information 13 [file peerj-12-16740-s013.zip › WB-Figure 5_CCLP1/Figure 5M_FAS_CCLP1.tif]

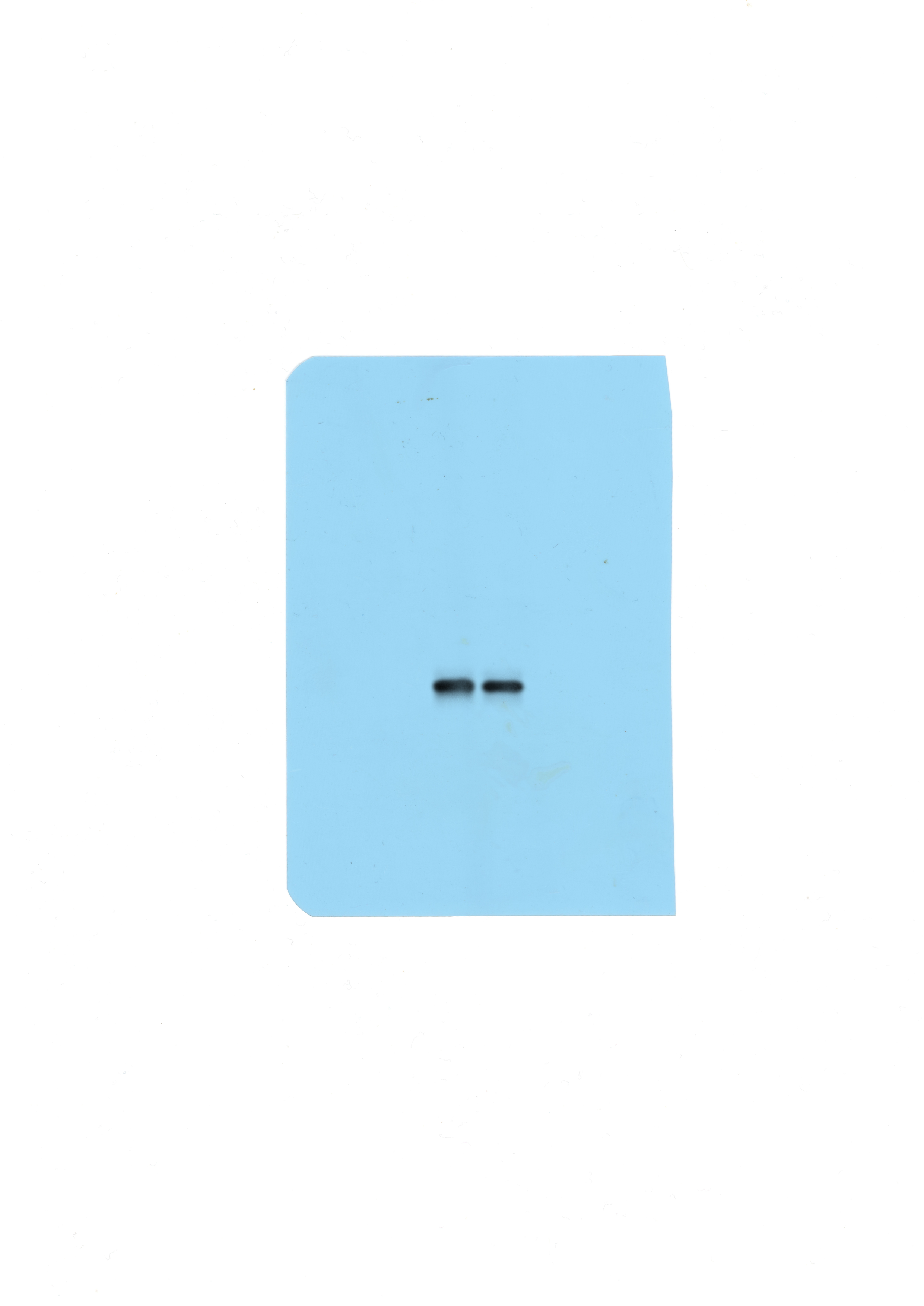

Supplement: Supplemental Information 13 [file peerj-12-16740-s013.zip › WB-Figure 5_CCLP1/Figure 5M_GAPDH_CCLP1.tif]

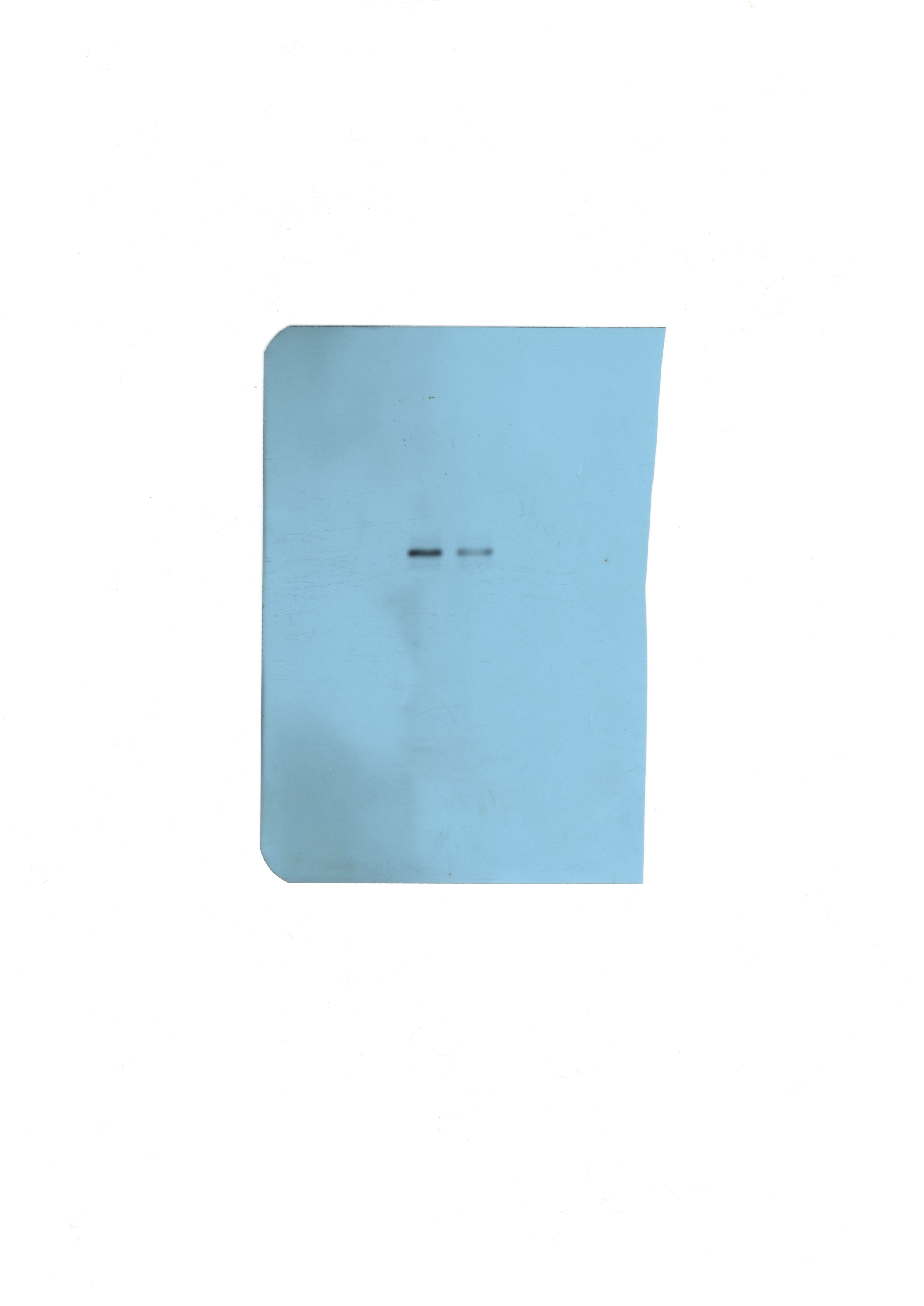

Supplement: Supplemental Information 13 [file peerj-12-16740-s013.zip › WB-Figure 5_CCLP1/Figure 5M_GPT-1_CCLP1.tif]

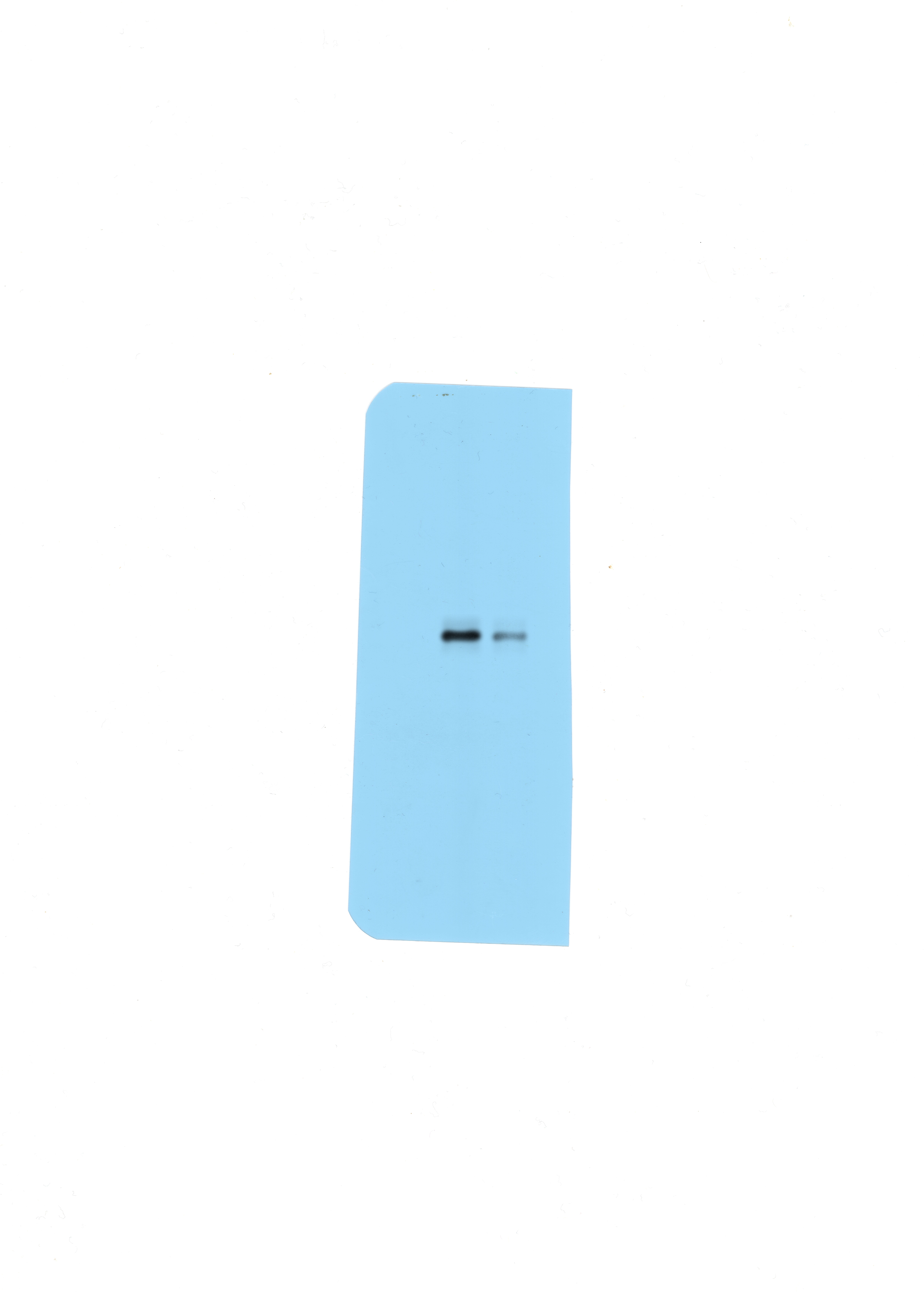

Supplement: Supplemental Information 13 [file peerj-12-16740-s013.zip › WB-Figure 5_CCLP1/Figure 5M_PPARa├_CCLP1.tif]

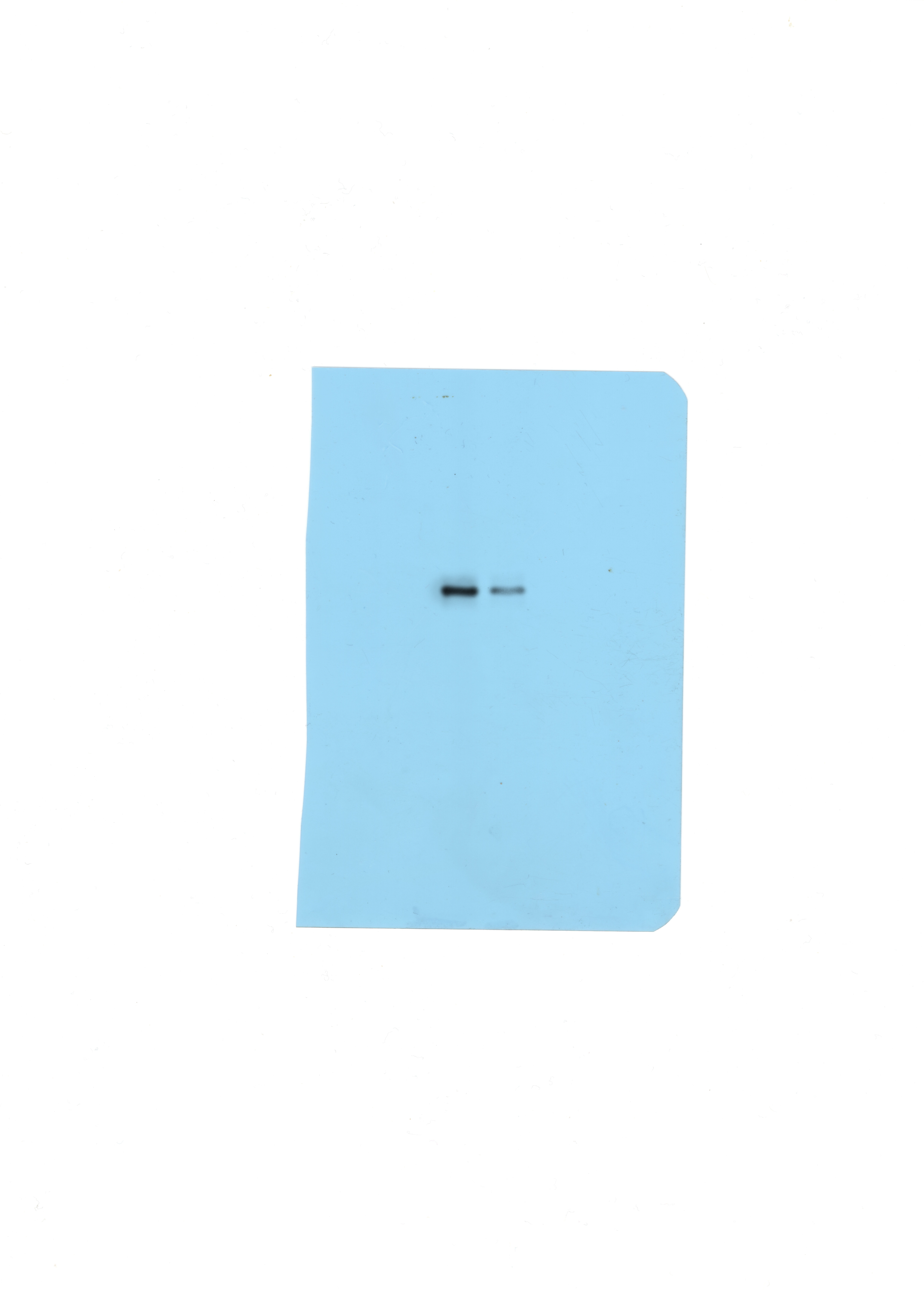

Supplement: Supplemental Information 13 [file peerj-12-16740-s013.zip › WB-Figure 5_CCLP1/Figure 5M_SCD1_CCLP1.tif]

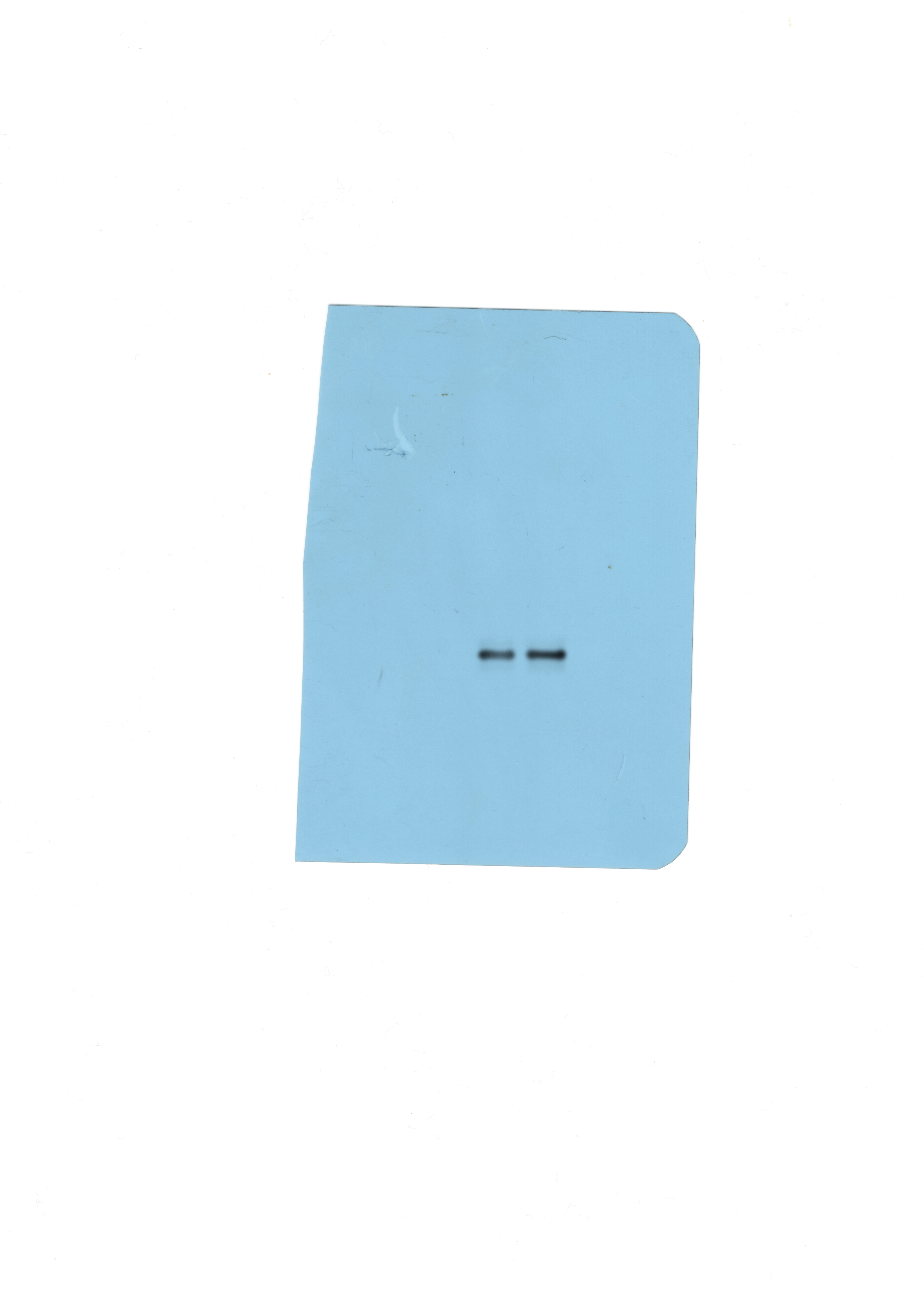

Supplement: Supplemental Information 13 [file peerj-12-16740-s013.zip › WB-Figure 5_CCLP1/Figure 5M_SREBP1_CCLP1.tif]

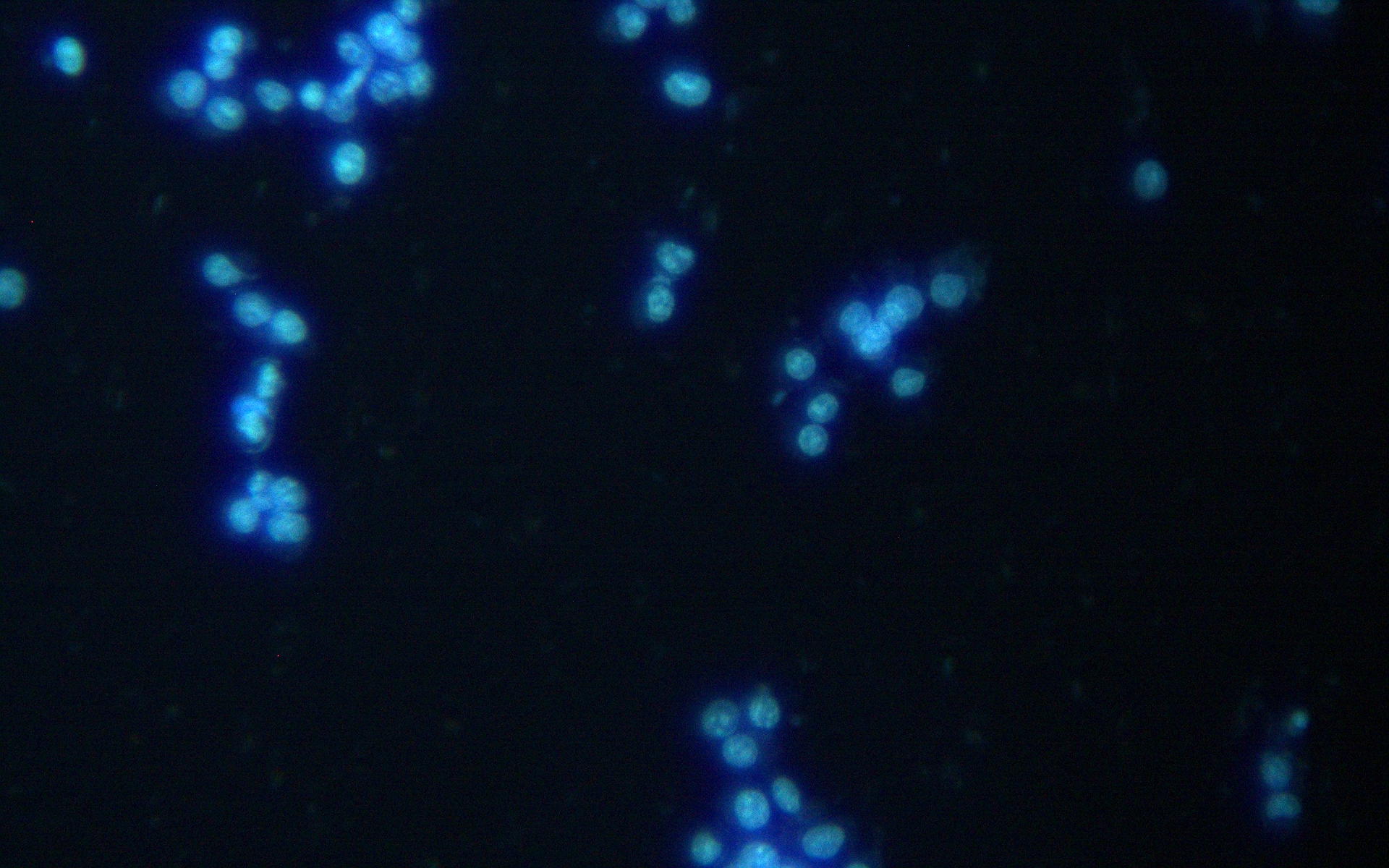

Supplement: Supplemental Information 14 [file peerj-12-16740-s014.zip › transwell-migration-HuCCT1/Figure 4H_over-APOE4_HuCCT1_Migration.tif]

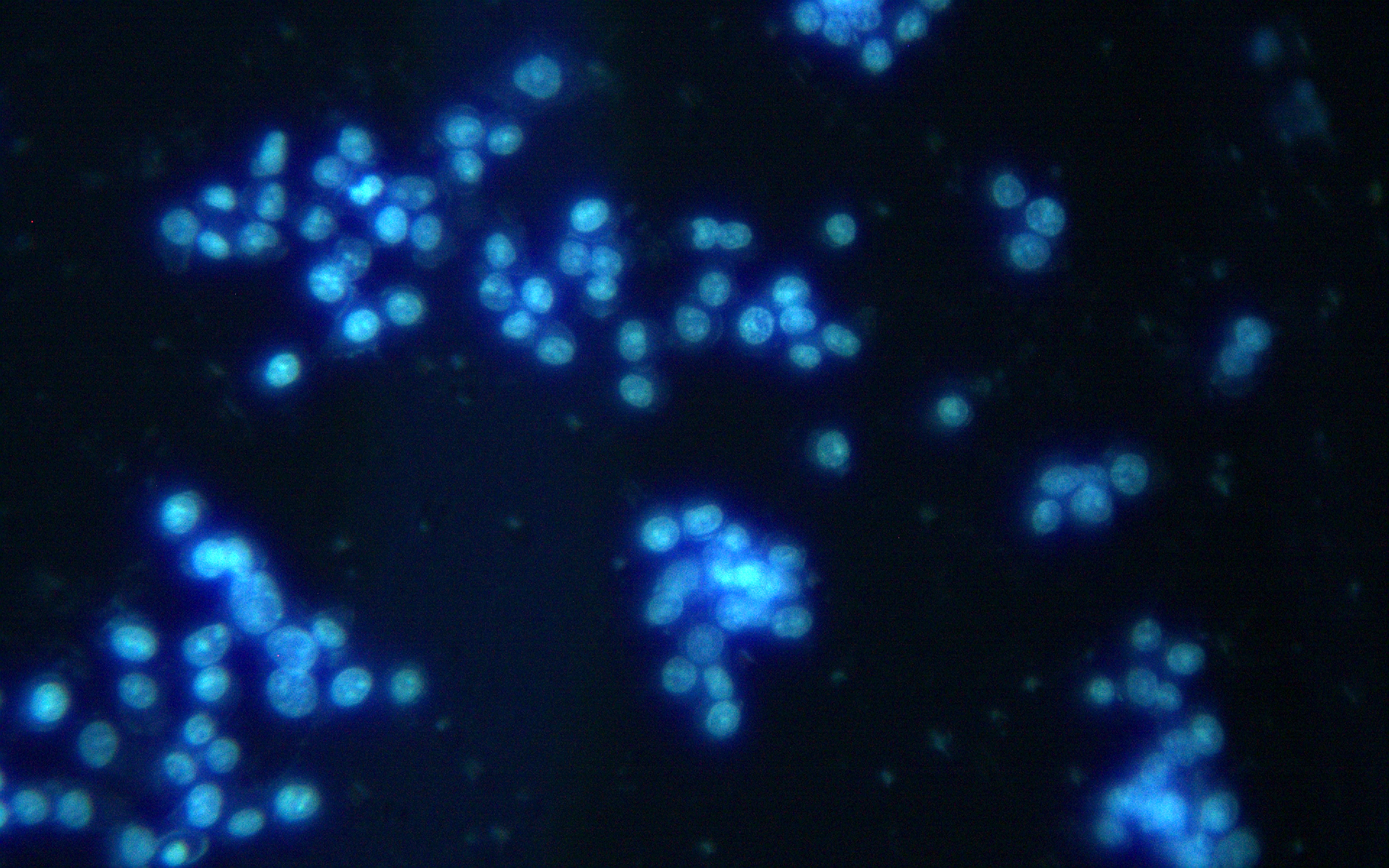

Supplement: Supplemental Information 14 [file peerj-12-16740-s014.zip › transwell-migration-HuCCT1/Figure 4H_over-NC_HuCCT1_Migration.tif]
