# Supplementary material for: Regulation of lipid metabolism by APOE4 in intrahepatic cholangiocarcinoma via the enhancement of ABCA1 membrane expression
Source: PeerJ. 2024 Jan 22;12:e16740. doi: 10.7717/peerj.16740 (PMC10809977; doi:10.7717/peerj.16740)
Supplement: Table S1 [file peerj-12-16740-s002.docx]

**Primer sequences of qRT-PCR**

| Primer | Sequence (5′-3′) |
| --- | --- |
| APOE4 | Forward: 5'-CAGTCTCTCACACTCGTCCTGGC-3'  Reverse: 5'-CTGCTCCTTCACCTCGTCCA-3' |
| FAS | Forward: 5′-CAAGAACTGCACGGAGGTGT-3′  Reverse: 5′-AGCAGCCAGAGTCGGAGAAC-3′ |
| ACC | Forward: 5′- CACGCTCAAGTCACCAAGAA-3′  Reverse: 5′- CCAAATGGGAGGCAATAAGA-3′ |
| SCD1 | Forward: 5′-GGCTCCTAGCCTAATCCCCT-3′  Reverse: 5′-CTTGCGATATGCTGTGGTGC-3′ |
| GPT-1 | Forward: 5′-TCTTGTCCTCAGTGCTTGGG-3′  Reverse: 5′-TGCGTCACAGCTGAAAGTGA-3′ |
| SREBP1 | Forward: 5′- AAACAGGGCATCACACACATC-3′  Reverse: 5′- GTGCTCGCAAAATGGCTGTA-3′ |
| PPARγ | Forward: 5′-CGTCCCCGCCTTATTATTCT-3′  Reverse: 5′-GCTTTATCCCCACAGACTCG-3′ |
| ABCA1 | Forward: 5′-CCCCTGTTTCCGTTACCC-3′  Reverse: 5′-AGCCCTCAGCATCTTGTC-3′ |
| GAPDH | Forward: 5′-GGACCAATACGACCAAATCCG-3′  Reverse: 5′-AGCCACATCGCTCAGACAC-3′ |
